# A description of interventions promoting healthier ready-to-eat meals (to eat in, to take away, or to be delivered) sold by specific food outlets in England: a systematic mapping and evidence synthesis

**DOI:** 10.1186/s12889-016-3980-2

**Published:** 2017-01-19

**Authors:** Frances C. Hillier-Brown, Carolyn D. Summerbell, Helen J. Moore, Wendy L. Wrieden, Jean Adams, Charles Abraham, Ashley Adamson, Vera Araújo-Soares, Martin White, Amelia A. Lake

**Affiliations:** 1 Obesity Related Behaviours Research Group, School of Medicine, Pharmacy & Health, Wolfson Research Institute, Durham University, Durham, UK; 2 Fuse – UKCRC Centre for Translational Research in Public Health, Newcastle University, Newcastle-upon-Tyne, UK; 3 Human Nutrition Research Centre, Institute of Health & Society, Newcastle University, Newcastle upon Tyne, UK; 4 Institute of Health & Society, Newcastle University, Newcastle upon Tyne, UK; 5 Psychology Applied to Heath, University of Exeter Medical School, University of Exeter, Exeter, UK; 6 Centre for Public Policy & Health, School of Medicine, Pharmacy & Health, Wolfson Research Institute, Durham University, Durham, UK; 7 Present address: CEDAR – UKCRC Centre for Diet and Activity Research, MRC Epidemiology Unit, University of Cambridge School of Clinical Medicine, Cambridge, UK

**Keywords:** Ready-to-eat-meals, Takeaways, Restaurants, Food environments, Diet, Nutrition, Obesity, Public health, Intervention, Evaluation

## Abstract

**Background:**

Ready-to-eat meals (to eat in, to take away or to be delivered) sold by food outlets are often more energy dense and nutrient poor compared with meals prepared at home, making them a reasonable target for public health intervention. The aim of the research presented in this paper was to systematically identify and describe interventions to promote healthier ready-to-eat meals (to eat in, to take away, or to be delivered) sold by specific food outlets in England.

**Methods:**

A systematic search and sift of the literature, followed by evidence mapping of relevant interventions, was conducted. Food outlets were included if they were located in England, were openly accessible to the public and, as their main business, sold ready-to-eat meals. Academic databases and grey literature were searched. Also, local authorities in England, topic experts, and key health professionals and workers were contacted. Two tiers of evidence synthesis took place: type, content and delivery of each intervention were summarised (Tier 1) and for those interventions that had been evaluated, a narrative synthesis was conducted (Tier 2).

**Results:**

A total of 75 interventions were identified, the most popular being awards. Businesses were more likely to engage with cost neutral interventions which offered imperceptible changes to price, palatability and portion size. Few interventions involved working upstream with suppliers of food, the generation of customer demand, the exploration of competition effects, and/or reducing portion sizes. Evaluations of interventions were generally limited in scope and of low methodological quality, and many were simple assessments of acceptability.

**Conclusions:**

Many interventions promoting healthier ready-to-eat meals (to eat in, to take away, or to be delivered) sold by specific food outlets in England are taking place; award-type interventions are the most common. Proprietors of food outlets in England that, as their main business, sell ready-to-eat meals, can be engaged in implementing interventions to promote healthier ready-to-eat-food. These proprietors are generally positive about such interventions, particularly when they are cost neutral and use a health by stealth approach.

**Electronic supplementary material:**

The online version of this article (doi:10.1186/s12889-016-3980-2) contains supplementary material, which is available to authorized users.

## Background

Ready-to-eat meals (to eat in, to take away, or to be delivered) sold by specific food outlets that, as their main business, sell ready-to-eat meals, are often more energy dense and nutrient poor compared with meals prepared and eaten at home [1]. Furthermore, the consumption of ready-to-eat meals sold by food outlets is associated with higher energy and fat, and lower micronutrient intake [2], and eating takeaway or fast food is associated with excess weight gain and obesity [3, 4].

The popularity and prevalence of eating ready-to-eat meals sold by food outlets has risen considerably over the last few decades in many high and middle income countries [5–7]. For example, around one fifth to one quarter of the UK population eat takeaway meals at home at least once per week [7]. There is some evidence that food outlets selling takeaway meals and fast foods are clustered in areas of deprivation [8]. Ready-to-eat meals sold by food outlets, particularly in deprived areas, are therefore a reasonable target for public health intervention [9].

A systematic review of the world literature on the impact of such interventions [10] identified only 13 interventions (12 in peer review publications), 11 of which were based in the US and one each in Canada and South Korea. The review found a limited range of practices that food outlets were asked to change as part of the intervention; all interventions included signage and labelling to promote healthful food options, several promoted more healthful cooking methods, and only one introduced new healthful menu options. The authors summarised the impact of these 13 interventions as being promising.

Since March 2011 the Department for Health (England), through the ‘Public Health Responsibility Deal’, has worked with a number of national and regional chain food outlets operating in England to promote healthier ready-to-eat meals. Chain food outlets ‘sign up’ to the nutrition guideline and pledge to implement a range of interventions to promote the sale of healthier ready-to-eat meals. Many of these interventions have used ‘health by stealth’ approaches, e.g. reformulation (particularly salt reduction, the removal of trans fats, and calorie reductions), and removing condiments from tables in sit-in eateries. Other interventions have focused on promoting smaller portion sizes (for example through re-packaging, or offering smaller options in addition to regular size meals), and providing consumers with better nutritional information (for example calorie labelling on menus) [11].

However, there are very few independently owned food outlets signed up to the Responsibility Deal despite the fact that there is a Local Responsibility Deal (see https://responsibilitydeal.dh.gov.uk/local-partners/ [12]) which the Department of Health (England) has been encouraging local authorities to promote to businesses in their area. This is of particular concern because the nutritional quality of food sold by independent food outlets is, in general, less healthy than that sold by chain food outlets [1]. Also, owners of these outlets, particularly those in deprived areas, appear to be less willing to engage in health-promoting interventions [13, 14]. A range of interventions are currently being championed by local government in England to promote healthier ready-to-eat foods sold by independent food outlets, but these tend to be poorly catalogued and described [15]. Indeed, our work with this review and others has shown that information on applied public health research questions relating to the nature and range of public health interventions, as well as many evaluations of these interventions, may be predominantly, or only, held in grey literature and grey information [16]. In addition, the evidence base around the development, implementation and effectiveness of these interventions is unclear and scattered. Together, these problems make it hard for those planning, designing and delivering new interventions to build on previous learning.

The research presented in this paper, and a related ‘sister’ review ([17, 18]), attempt to fill these evidence gaps. Our related ‘sister’ review found that the evidence is dominated by interventions in national and multinational chain food outlets operating in North America; only one intervention from the UK was identified. This ‘sister’ review of the effectiveness of such interventions was restricted to evaluations of interventions which include an assessment of impact/outcome that were conducted anywhere in the world, identified through academic database searches and published in peer review publications. In contrast, the paper reported here includes a description of relevant interventions in England and, where available, evaluations of interventions which include an assessment of process, acceptability, cost, and/or impact/outcome conducted, identified through academic database and grey literature searches and information from various contacts.

The aim of the research presented in the current paper, therefore, was to systematically identify interventions to promote healthier ready-to-eat meals (to eat in, to take away, or to be delivered) sold by specific food outlets in England. Where possible, we aimed to describe the type of interventions, and summarise information on their content and delivery. In addition, for those interventions which had been evaluated, we aimed to summarise information from these evaluations.

## Methods

We conducted a systematic search and mapping of the evidence, and an evidence synthesis, using methods adapted from standard systematic review techniques [19, 20], of interventions to promote healthy ready-to-eat meals (to eat in, to take away, or to be delivered) sold by specific food outlets in England.

### Inclusion criteria

The specific food outlets we included were those that, as their main business, sold ready-to-eat meals and beverages, and were openly accessible to the general public. Supermarkets and general food stores selling ready-to-eat meals (e.g. salad boxes and sandwiches) were not included, but cafes and restaurants within supermarkets and other retail stores selling ready-to-eat meals were. Food outlets which would otherwise meet the inclusion criteria, but provided ready-to-eat meals free of charge (e.g. community based lunch clubs for the elderly or homeless), were excluded. We also excluded food outlets which are not openly accessible to the general public, including those based in schools and universities, workplaces, and health or social care institutions: the effects of interventions to promote the sale of healthier meals in these food environments has previously been reviewed, e.g. [21–23].

We did not specifically exclude food outlets where the only option was to eat in, and as such we ran the risk of including interventions targeted at ‘high end’ restaurants.

The categorisation of types of food outlets to be included was developed using previous work on this topic area by Lake et al. [24, 25]. This work identified various categories of food outlets, of which nine were deemed relevant for this review (see Additional file 1). Food outlets targeted by the interventions included in this review were mapped onto these 9 categories of food outlets; some food outlets mapped onto more than one category.

Our knowledge of the evidence base in this area comes from our sister review [18], where after searching the bibliographic databases we identified just one uncontrolled study conducted in England [26] (included in this article as Award 34). Given the aim of the present review was to provide an inclusive and comprehensive list and description of relevant interventions, we did not set any inclusion criteria based on how or where information about relevant interventions (or evaluations of them) was reported, or methodological quality of this information. For example, we considered assessments of acceptability of the intervention (by the project team, the food proprietor and staff, or the customer) as evaluations for the purpose of this review.

### Systematic search and mapping

Bibliographic databases, research and trial registers, and grey literature, were searched for relevant information between December 2013 and January 2014 (by FHB and HJM); see Table 1 for more information. In addition, between January and March 2014, a list of people were contacted (via social media, email, routine newsletters, magazines, bulletins and websites, by FHB) asking for relevant information. These included key contacts in all 353 local authorities in England, topic experts, and relevant health professionals and workers; see Additional file 2 for more information.

**Table 1:** Academic and grey literature searches and search terms used to identify interventions to promote healthier ready-to-eat meals (to eat in, take away, or delivered) sold by specific food outlets in England

Academic searches	
Bibliographic databases	MEDLINE (Ovid), EMBASE (Ovid), CINAHL (Ebscohost), PsycINFO (Ebscohost), ASSIA (ProQuest) and the NHS Economic Evaluation Database (Wiley Cochrane). (searched from start 1993 to end 2013). For more details about search strategies, please see references [17, 18]
Research and trial Registers^a^	The National Research Register (NRR) (archived from 2000 to 2007) and the International Standard Randomised Controlled Trial Number (ISRCTN) Register (search date 10 January 2014)
Grey literature searches^a^	
Grey literature databases	OpenGrey, Social Care Online and Prevention Information & Evidence eLibrary (search date 16 December 2013)
Media database	Nexis (search date 16 December 2013)
Specific websites	Food Standards Agency (archived web site from 2001 to 2009), Department of Health, Public Health England, National Obesity Observatory, Chartered Institute of Environmental Health (CIEH), Food Vision, Change4Life, Sustain, British Heart Foundation, Obesity Learning Centre, UK Health Forum, NICE, Food For Life, Soil Association, Focus On Food Campaign, RH Environmental, Children’s Food Trust and Local Food Grants (searches conducted 13–16 January 2014).
Internet search engine^b^	Google (searches conducted 17–23 December 2013)

^a^Search terms used for research and trial registers, and grey literature searches, were: Fast food, take-away, out-of-home food, café, restaurant, food environment, health, healthy eating, programme, project, intervention

^b^The first 100 hits of each search were accessed, or earlier if saturation was achieved (i.e. no new interventions were found in the last 20 hits)

All bibliographic and grey literature searches were performed by FHB or HJM. All search results from the academic literature were screened for relevance by FHB, AAL, HJM or CDS. All search results from the grey literature were screened for relevance by FHB. Responses to information requests were screened for relevance by FHB. Any instances of uncertainty were resolved through discussion with AAL.

**Fig. 1:**
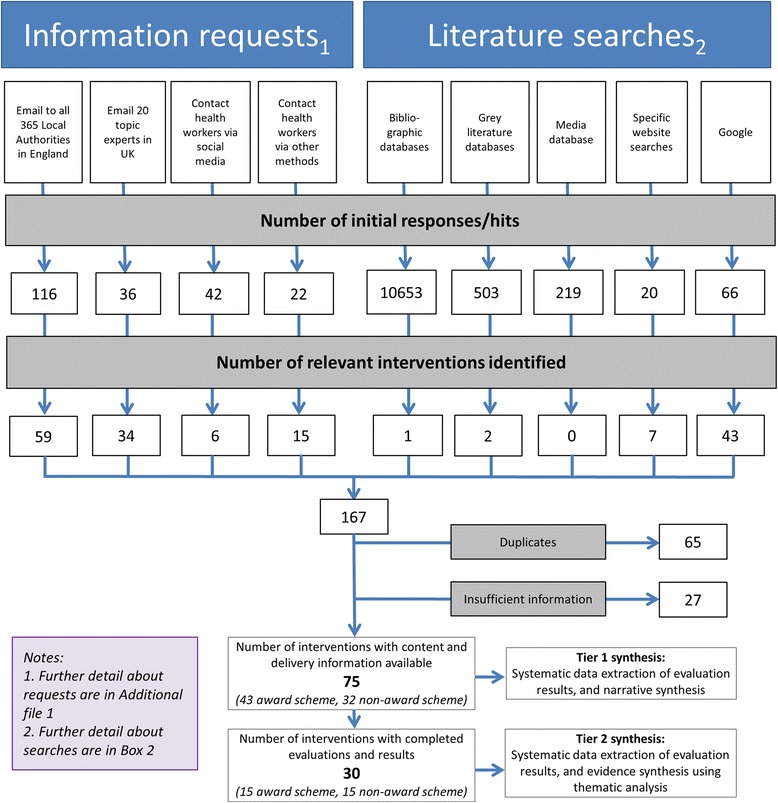
Systematic search and mapping of interventions to promote healthier ready-to-eat meals (to eat in, take away, or delivered) sold by specific food outlets in England: flow diagram

Given that information about some interventions was reported from more than one source (Fig. 1), in different formats and by different people, a careful mapping of interventions was conducted by FHB. Areas of uncertainly were resolved through discussion with AAL. Information on the name, location, type, aim and description of the intervention, and the intervention team, was extracted for each intervention. For data extraction, we developed, piloted, and used a data extraction pro forma. Where we had just a small amount of information, for example from an email correspondence or a brief article on a website, we chose to include all available information. Data extractions were conducted by FHB, AAL, CDS or WLW and checked by FHB and AAL. Any discrepancies were resolved by CDS.

### Evidence synthesis

Two tiers of evidence synthesis took place, depending on data availability. Where enough information was available to assess the type, content and delivery of the intervention (Tier 1), this information was systematically extracted onto a pro forma, and details were sent to the relevant contacts to check for accuracy and completeness. Examples of ‘enough information’ in this context were *‘calorie labelling and reformulation’ (Non-award intervention, No 11)* for content, and *‘information was provided to the food outlet’ (Non-award intervention, No 2)* for delivery. A summary of this information is presented as a narrative synthesis.

Where interventions had been evaluated, regardless of the extent or methodological quality of the evaluation (Tier 2), information on the design, methods and results of these evaluations were also extracted onto the pro forma and details sent to the relevant contacts to check for accuracy and completeness. A summary of this information is presented in Table 2 in this paper, and a narrative synthesis is presented.

**Table 2:** Summary of the evaluations of interventions to promote healthier ready-to-eat meals (to eat in, take away, or delivered) sold by specific^a^ food outlets in England (Tier 2, *n* = 30)

Project name (reference number)	Type of food outlet targeted by the intervention^b^, and notes^c^	Process	Acceptability			Cost			Impact/Outcome			Comments related to food outlets 1) working upstream (*n* = 6), 2) favouring a health by stealth approach (*n* = 10), and 3) generating customer demand (*n* = 3), and other information.
		Description	++ favourable, + favourable overall but included some negative aspects, 0 ambivalent, − negative overall but included some positive aspects, −- negative									
		Food outlet	Project team	Food outlet	Customer	Project team	Food outlet	Customer	Project team	Food outlet	Customer	
Rochdale Borough Council’s Healthier Chips (Award 2)	Takeaway eateries (1) Notes: specifically outlets near schools			**++**	**+**							
The Cornwall Healthier Eating and Food Safety (CHEFS) Award (Award 6)	Takeaways and Sit in eateries (1, 2 and 3)			**+**								*Upstream issues*: difficulties sourcing alternative food products
Kirklees Healthy Choice Award (Award 10)	Takeaways and Sit in eateries (1, 2 and 3)	Only one business chose not to renew their award		**+**								
Recipe4Health, Lancashire (Award 15)	Takeaways and Sit in eateries (1, 2 and 3)			**++**			**++**			**++**		Cost and impact/outcome results based on 1–2 case studies
Tower Hamlets Healthy Towns/Healthy Food Award/Food for Health (Award 20)	Takeaways and Sit in eateries (1, 2 and 3)			**+**			**0**					*Health by stealth:* Most businesses found changing to a healthier oil was the easiest criteria to meet
Bristol Better Sandwiches project (Award 25)	Takeaway eateries (1) Notes: independent outlets only (*n* = 20 outlets at baseline)	At 3 years: 4 closed down, 3 changed hands & 13 were still trading as the same business. Some staff changes and new managers resulting in little memory of the intervention.		**-**			**0**			**++**		The negative view around acceptability was focussed on the fact that the resource for the intervention had ended.
Heartbeat Award (Health Education Authority), England-wide (Award 26) [40, 41]	Takeaways and Sit in eateries (1, 2 and 3) Notes: intervention aimed at lower SES groups				**+**					**++**		*Generation of customer demand*: the majority of respondents agreed that healthy food choices should be available when eating out. *Health by stealth:* Award premises purchased significantly more brown rice and semi/skimmed milk, and skinned chicken before cooking.
Eat Well Award, Undisclosed PCT in the North West (Award 27) [42]	Takeaways and Sit in eateries (1, 2 and 3) Notes: outlets in disadvantaged areas		**-**									
Healthy Business Award, Ashton, Leigh, Wigan (Award 29)	Takeaways and Sit in eateries (1, 2 and 3) Notes: included outlets in deprived areas									**++**	**+**	*Generation of customer demand:* 54% of which customers said they were positively influenced by the fact it was a ‘Healthier Choice’
Healthier Options Food Awards, Newham (Award 30)	Takeaways and Sit in eateries (1, 2 and 3)										**+**	
London Healthier Catering Commitment (overall) (Award 34) (HCC) [26]	Takeaways and Sit in eateries (1, 2 and 3) Notes: included outlets in deprived areas			**-**						**+**		*Upstream issues:* Difficulties sourcing low fat products from existing suppliers *Health by Stealth:* Businesses reported fearing that customers would not like the taste of food cooked without any salt
London Healthier Catering Commitment, Hammersmith and Fulham, Kensington and Chelsea and Westminster (Award 40) (HCC)	Takeaways and Sit in eateries (1, 2 and 3) Notes: Outlets in affluent and deprived areas		**+**	**+**	**0**		**0**			**+**		*Health by stealth:* businesses appreciated the fact that the changes required of them were fairly minor. Changes made to the use of oil and salt were adopted by the largest number of businesses.
London Healthy Catering Commitment, Sutton and Merton (incorporated in Sutton and Merton Responsibility Deal) (Award 41) (HCC)	Takeaways and Sit in eateries (1, 2 and 3) Notes: independent outlets			**+**						**+**		*Generation of customer demand:* 43% of 42 business said they are selling more water and diet drinks now they are prominently displayed; 14% of the businesses reported their customers have been asking for smaller portions now they are clearly advertised
London Healthy Catering Commitment, London Borough of Richmond (Whitton & Heathfield) (Award 42) (HCC)	Takeaways and Sit in eateries (1, 2 and 3) Notes: independent outlets			**+**	**+**					**+**		
London Healthy Catering Commitment, London Borough of Richmond (Ham, Sheen and Twickenham) (Award 43) (HCC)	Takeaways and Sit in eateries (1, 2 and 3) Notes: outlets near schools	23 out of 60 achieved award. 17 of 37 restaurants and cafes achieved award, compared with 6 of 23 takeaways.		**0**								Negative views of acceptability expressed by takeaways compared with restaurants and cafes.
Eatright Liverpool (Non-award 9)	Takeaways and Sit in eateries (1, 2 and 3)	Trust between the takeaways and support team essential to project. Takeaways, do not document recipes. Some dietary analysis software inappropriate										
Worcestershire Truckers Tucker (Non-award 15)	Takeaways and Sit in eateries (1, 2 and 3)			**+**	**+**		**+**			**+**		*Health by stealth:* Top Tips successfully implemented included using healthier products and cooking practices, of which the customer would be unaware.
Central England Trading Association Truckers Tucker (Non-award 16)	Takeaways and Sit in eateries (1, 2 and 3)		**+**	**+**	**+**		**0**			**0**		Impact/outcome based on 2 cases *Health by stealth:* proprietors top tips included changes which their customers (in all except one premise) did not notice any difference in taste. Top Tips easiest to implement included using healthier products and cooking practices, of which the customer would be unaware.
Shropshire Eat Well live Longer - on the road (Non-award 17)	Takeaways and Sit in eateries (1, 2 and 3) Notes: outlets in areas of social deprivation			**+**			**+**			**++**		*Upstream issues*: Specific healthier products are not always available in wholesalers. *Health by stealth:* Businesses successfully implemented the use of healthier products and cooking practices, of which the customer would be unaware.
Warwickshire Truckers Tucker (Non-award 18)	Takeaways and Sit in eateries (1, 2 and 3)			**++**								
Healthier menu choices for children, South Somerset (Non-award 21)	Takeaways and Sit in eateries (1, 2 and 3) Notes: independent outlets			**+**						**+**		Acceptability views by food outlets limited to their views on the training provided
Box chicken, London (Non-award 23)	Takeaway eateries (1) Notes: outlets near schools, particularly in low income areas				**++**					**+**		
Enfield healthier takeaways project (Non-award 24)	Takeaway eateries (1)			**-**	**-**					**+**		
Stoke-on-Trent Chip shop project (Non-award 25)	Takeaway eateries (1)									**+**		*Health by stealth:* Businesses successfully implemented the use of healthier products and cooking practices, of which the customer would be unaware.
Shake Less Salt campaign, Norfolk (Non-award 26)	Takeaway eateries (1)			**+**	**-**		**+**			**+**		*Health by stealth*: findings suggest customers favour a ‘health by stealth’ approach.
Gateshead Salt Shakers (Non-award 27)	Takeaway eateries (1)	Only 3 businesses approached declined to take part. A large proportion of shops agreed to provide a poster and leaflets.		**++**			**+**			**+**		Cost and impact/outcome results based on one case
Sandwich project, Exeter (Non-award 28)	Takeaway eateries (1)			**++**			**++**			**++**		*Health by stealth:* Businesses successfully implemented the use of healthier products, of which the customer would be unaware.
Sandwich project, Buckinghamshire (Non-award 29)	Takeaway eateries (1)		**+**							**+**		
My Choice, London (Non-award 30)	Takeaways and Sit in eateries (1, 2 and 3) Notes: outlets in a deprived area				**+**							
FSA project - calorie information at the point of choice in catering outlets, UK wide (Non-award 31)	Takeaways and Sit in eateries (1, 2 and 3)			**+**	**+**						**0**	

^a^The specific food outlets included were those that, as their main business, sold ready-to-eat meals and were openly accessible to the general public

^b^Food outlets targeted by the intervention were mapped (see Additional file 1 for detail of process) onto one of three categories:

1. Takeaway eateries (takeaways)

2. Sit-in eateries

3. Food outlets that included options to takeaway or sit-in

^c^Information on whether the intervention included chain and/or independent outlets, and/or had a particular focus on low SES groups or outlets near schools, where reported

## Results

The systematic search and mapping identified 75 relevant interventions, and these were included in the Tier 1 synthesis (Fig. 1) and are listed in Additional file 3. For completeness, interventions we identified that sounded relevant from their titles, but were excluded because there was insufficient information to assess the type, content and delivery of the intervention, are listed in Additional file 4. Data collected for the Tier 1 evidence synthesis are reported in Additional file 5 and summarised in Additional file 6.

### Type of interventions

The single distinguishing factor around which interventions could be reasonably categorised was whether or not they were awards. ‘Award’ type interventions were defined as those that involved an assessment of food outlet practice(s) targeted by the intervention using pre-defined criteria, together with some sort of accreditation if the food outlet met the criteria. Of the 75 interventions, 43 were awards of which 14 were based on the Charted Institute of Environmental Health‘s Healthier Catering Commitment (HCC) for London [27]. The remaining 32 non-award interventions were heterogeneous in nature.

### Nutrient/food group targets

This information is provided in Additional file 5, under aims or intervention description. Awards often included multiple nutrient targets for change and assessment of intervention success (e.g. fat, salt, and sugar content of meals on sale) and usually had levels of award (e.g. bronze, silver, gold). In contrast, most ‘non-award’ interventions focused on changing specific nutrients (e.g. salt or fat). Awards usually targeted a broad range of food outlets, whereas most non-award interventions focused on specific types of food outlets (e.g. Fish and chip shops or sandwich shops).

### Project funding

Information about funding for the projects team, and associated intervention costs for the food outlet proprietor, and sustainability of this funding, was available for 18 interventions (data not reported). Funding was usually described as being time-limited, and sourced from existing local government budgets. Although the available information is limited, sustainable funding routes appear uncommon.

### Intervention delivery costs for the food outlets

Some information on set up and running costs was provided for a third (*n* = 25) of the interventions and eight provided detailed values. This information is not reported in detail here due to its sensitive nature. Where details were provided, the delivery of most interventions was reported as being cost neutral to the food outlet businesses.

### Type and location of food outlet targeted

Forty-nine of 75 interventions were not targeted at any specific type of food outlet, and 24 were targeted at takeaways only. One intervention was targeted at an independent café that primarily offered an eat in option. Another intervention was targeted at the eat in aspect of food outlets which could be considered as low to reasonable cost, fast service cafes, restaurants and pubs (for example Jamie’s Italian, Nando’s, Frankie and Benny’s, McDonald’s and Weatherspoons). These two interventions were classified as sit-in eateries for the purpose of this review. In seven cases it was clear that interventions were specifically targeted at independent food outlets. Thirteen interventions were targeted at food outlets in deprived areas, and seven interventions were targeted at food outlets very close to schools.

### Project teams

This information is provided in Additional file 5, under details of intervention team, expertise and award accredited by. The majority (54 of 75) of project teams involved in the promotion of the intervention to the food outlets were local government environmental health officers in partnership with other professionals. These included: trading standards staff, public health professionals, dietitians and community nutritionists. Awards were mostly accredited by local government environmental health, food safety and/or trading standards officers. Twenty-one (of 75) project teams were non-governmental organisations, independent nutritionists, or ‘not for profit’ organisations.

### Description of support provided by the project team to the food outlets proprietors and their staff

A key feature of award type interventions was, as expected, the process of accreditation by the project teams (all 43). For many interventions (48 of 75), particularly award type interventions, one assessment at a single point in time of the food outlet practices by the project team against a pre-determined criteria was conducted. In practice, this involved the food outlet signing up to the intervention, then in some cases (32 of 48) being sent or signposted to relevant support information, and then assessed by the project team. The re-assessment of practices post intervention was only clearly reported in one award-type intervention and five non-award type interventions.

Support provided included standard leaflets or booklets, (*n* = 31), personalised support or feedback for the staff and proprietor (*n* = 28), training for the staff and proprietor (*n* = 15), and equipment provision (*n* = 11). Few interventions involved the project team working upstream with suppliers of food to the food outlet (*n* = 6), for example to enable the businesses to source equipment or healthier ingredients which they could use as alternatives (e.g. low-fat mayonnaise, low-fat spread, a different type of cooking oil), or generating customer demand (*n* = 2). By generation of customer demand, in this context, we mean the process by which project teams create or reinforce customer desire for healthier food options through education and/or encourage or support customers to ask for healthier options in food outlets so that this desire is communicated.

We did not identify any evidence of project teams working with businesses to encourage them to provide healthier ready-to-eat meals through the creation of competition with other food outlets, but we did find one intervention where the effects of competition were explored by the project team [Non-award 20]. By competition, in this context, we mean the process by which food outlets could market the healthier ready-to-eat meals on their menus as a competitive advantage in comparison with the (less healthy) options available from their direct competitors. These marketing strategies are commonly used in business [28], and have been used as part of interventions to increase the sale of healthier food [29].

### Description of the practices that food outlets were asked to change as part of the intervention

The most common practice targeted by interventions was adapting existing cooking practices, including recipe reformulation and changing ingredients used (in 45 of 75 interventions). The removal of ‘unhealthy options’ was only clearly reported in seven interventions, but adding ‘healthier’ food or drink options, for example fruits and vegetables, low or no sugar drinks, and smaller portion size options alongside regular portions, was clearly reported in about half of cases (*n* = 37). Marketing and promoting healthy options, or that the business was participating in health promotion interventions, was reported in 26 interventions. Eighteen interventions included a focus on providing suitable options for children. Sixteen interventions clearly reported using menu labelling.

Six interventions clearly reported targeting reductions in portion size. Nine interventions included the provision of verbal or printed information for customers, above and beyond generic information included in the menus.

### Intervention evaluation

Thirty interventions were included in the Tier 2 synthesis (results shown in Additional file 7, and summarised in Table 2). The 30 evaluations included an assessment of the 1) process, 2) acceptability, 3) cost and/or 4) impact/outcome of the interventions. These assessments were focussed on the project team, the food outlet, and/or the customer. We also included a note of whether the evaluation included any information about issues relating to working upstream with suppliers, favouring a health by stealth approach, and the generation of customer demand.

### Evaluation study design

Sixteen of the 30 evaluations included post-intervention assessment only, and two only included pre-intervention assessment (e.g. baseline information on interest, and perceptions of acceptability and feasibility, of the intervention by the food outlet proprietor). Ten evaluations included a pre- and post-intervention assessment. Two evaluations included a control group: one including post-intervention assessments only [Award 26], and one both pre- and post-assessments [Non-award 28]).

### Evaluation methods

Overall, the methods used to collect data were poorly described but appeared mainly qualitative. Most evaluations collected information about the experiences and perceptions of the food outlet proprietors of interventions. Some also collected information on customer and the project team’s views about the intervention. Data was most commonly collected through surveys using postal questionnaires which were designed by the project teams. Face to face or telephone interviews were used in some evaluations, often as part of feedback and follow-up visits, and a focus group (with customers) was used in one evaluation [Non-award 31].

Fifteen of the 30 evaluations were of award-type interventions, of which five were based on the HCC [27]. Six of the 30 evaluations were of interventions targeted at take-away food outlets, three at food outlets near schools, four at independent food outlets, and seven at food outlets in areas of deprivation.

### Evaluation findings

**Process (** ***n*** **= 5):** Five evaluations included an assessment of process.

*Difficulties in assessing nutritional composition of foods served:* One evaluation [Non-award 9] that planned to assess the effect of interventions on nutritional composition of food sold highlighted a number of problems. Takeaway outlets, particularly independently owned food outlets serving predominately Chinese and Indian dishes, do not commonly document recipes. Even when recipes are documented, the absence of many ingredients from popular nutritional analysis software packages meant that the nutritional composition of dishes (and any changes, as a result of the intervention) could not be determined. Although laboratory based analysis of dishes are possible and attractive to local authorities, they were prohibitively expensive in many cases.

Process issues perceived by food outlet proprietors primarily stemmed from underlying concerns that interventions would have negative effects on the acceptability of food for their customers, and sales. One evaluation [Award 25] of interventions in independent takeaway food outlets highlighted the relatively high turnover of staff working in these outlets which resulted in limited and patchy knowledge of the intervention.

**Acceptability (** ***n*** **= 26)**: Twenty six evaluations included an assessment of the acceptability of the intervention; four from the perspective of the project team, 21 from the perspective of the food outlets, and 11 from the perspective of the customers.

***From the perspective of the project team*** *,* the acceptability and success of the intervention was, in part, dependent on project team’s skills and knowledge. The project team’s ability to be both positive and enthusiastic about the intervention, and their personal interest in healthier lifestyles, were deemed to be important factors. The ability of the project team to build rapport and trusting relationships with food outlet proprietors was also considered important for success. Promoting the intervention to food outlet proprietors and their staff, to the point where they agreed to take part, often required a higher time commitment than originally planned. Evaluations highlighted the perceived need for multi-disciplinary approaches; in most cases this meant the inclusion of a qualified nutritionist or dietitian, in addition to environmental health officers, in the project team. The evaluation team for one intervention [Award 27] perceived the fact that including a former chef, who had worked in a similar type of food outlet to the ones targeted, in the project team was key to the success of the intervention.

***From the perspective of the food outlet owners, managers and staff members*** *,* most (17 of 21) were positive about interventions. Overall, they particularly favoured interventions that did not affect the cost, palatability or portion size of the food served, and those which they felt were the easiest to implement. For example, mobile roadside cafés [Non-awards 15, 16 and 17] and a sandwich shop intervention [Non-award 28] reported that the changes to practice they found easiest to implement (and liked very much) were using healthier versions of standard ingredients (e.g. lower fat mayonnaise or spread) and using healthier cooking practices (e.g. draining food on kitchen roll before service; removing visible fat from bacon).

Two evaluations of interventions [Awards 6 and 41] found that food outlet proprietors reported benefits to staff health and knowledge. Also, two evaluations of interventions [Awards 6 and 10] found that food outlets perceived value in the public recognition associated with awards, which they thought improved customer satisfaction and confidence as well as attracting more customers.

One evaluation [Award 6] reported that food outlet proprietors raised initial concerns about food wastage as a result of adding healthier alternatives to their menus, and these then failing to sell. However, two other evaluations [Award 15 and Non-Award 28] experienced a decrease in waste in practice. Also, one evaluation [Award 6] reported that businesses had difficulties in training staff in new cooking and food preparation techniques.

One evaluation concluded that the intervention [Award 43] was acceptable in restaurants and cafes, but not takeaways, and three evaluations concluded that, overall, the intervention [Awards 25 and 34, and Non-award 24] was not acceptable to the food outlets. The main criticism around Award 25 was that this intervention had come to an end; for Award 34 the criticisms focussed on those changes which were perceptible to the customer, and for Non-award 24 the criticisms focussed around the use of the new 5-hole salt shaker which had resulted in customers taking longer to salt their food and increased queues in their outlets.

***From the perspective of the customers*** interviewed for eight of the 11 evaluations, they were in favour, overall, of the intervention, and particularly liked the increase in choice of healthier options’. However*,* in some cases [Awards 26 and 42, and Non-award 31] customers appeared to lack awareness of intervention, regardless of whether or not they were publicised. In one evaluation, some customers complained about the intervention [Award 2] along the lines of a ‘nanny state’.

One evaluation [Award 40] reported that customers did not feel that the intervention would make any difference to what they bought from the food outlet, and two evaluations [Non-awards 24 and 26] received negative views about the interventions from customers. In both cases, the intervention was a 5-hole salt shaker; some customers complained about the ‘lack of taste’ and longer queues due to it taking longer for customers to salt their food.

**Overall**, there was not enough information to determine if certain types of food outlets were more willing to participate in interventions. However, two evaluations contacted businesses who had *not* taken part in interventions [Award 20 and Award 26]. Reasons for not taking part included lack of time and interest in receiving an award, lost or unreceived invitations to take part, and too much concern about the potential effect of interventions on food palatability and sales. One evaluation [Award 27] reported that food outlets in deprived areas found it particularly challenging to generate profits and that interventions and project teams had to be sensitive this.

There was also not enough information to determine whether interventions were more effective in some type of food outlets compared with others. However, one evaluation of an award [Award 43] reported that engagement by restaurants, sandwich shops and cafes was higher than by takeaways, for two reasons. First, because the former typically did not have to make substantive changes to achieve award criteria, or the criteria (e.g. focusing on frying practice) were not relevant. Second, takeaways, where more frying took place, were often reluctant to change frying practices due to concerns about the potential impact on food palatability.

**Cost (** ***n*** **= 10):** Ten evaluations included an assessment of the cost of the intervention, all of which were from the perspective of the food outlets. Six food outlets reported an increase in profits and four food outlets reported no change. One evaluation of an intervention targeting mobile food outlets [Non-award 16] reported a saving in oil used due to the use of the small oil spray bottle for frying which was provided by the project team. Another evaluation of a 5-hole salt shaker intervention [Non-award 27] reported a saving in salt used.

**Impact/outcome (** ***n*** **= 21):** Twenty one evaluations included an assessment of the impact/outcome of the intervention; none from the perspective of the project team, 19 from the perspective of the food outlets, and three from the perspective of the customers.

Eighteen of the 19 evaluations found that the interventions had a positive impact from the perspective of the food outlet; one evaluation [Non award 16] found negligible impact. The project team who evaluated Non award 16 conducted nutrition sampling and analysis of meals offered by two of the food outlets involved in the intervention. In one case they found that the reduction in fat content of fried food was offset against larger portions being served. In another case, the only change that had been implemented was the use of wholemeal bread for white bread.

The positive impact reported in 18 of the evaluations related to the practices that food outlets were asked to change as part of the intervention (as listed in Additional file 6). Although a little unclear overall, it appears that certain practices which took a health by steal approach were more commonly implemented (see below).

One evaluation of an intervention that targeted independent takeaway food outlets [Award 25] included long term (3 year) follow up results. Challenges associated with a relatively high turnover rate of businesses, and staff working in food outlets, were identified. Although many of the staff reported little memory of the intervention at follow-up, all of the businesses still trading under the same owner at 3 years (80%) had sustained at least some of the changes made as a result of the intervention.

Two of the interventions [Awards 29 and 30] were perceived to have had a positive impact from the perspective of the customers, particularly in terms of their awareness and purchasing of meals that had been identified as ‘Healthier choices’ on the menu. One intervention [Non-award 31] which focussed on calorie labelling was perceived to have had a negligible impact because many of the customers struggled with, and didn’t appreciate, the calories labelling.

**Working upstream with suppliers** (***n*** = 3): Three businesses reported experiencing difficulties sourcing healthier ingredients and foods from suppliers. One business specifically reported difficulties sourcing lower fat spreads and mayonnaise [Award 34], and another business had similar difficulties sourcing tinned tuna in spring water (Non-award 17).

**Favouring a health by stealth approach** (*n*= 10): Ten businesses reported favouring a health by stealth approach to interventions. In general, they found that changing ‘like-for-like’ more acceptable compared with changes that would be more perceptible to the customer. Specific examples mentioned included using lower fat spread or lower fat mayonnaise for their full fat alternatives, using a healthier oil, and using a 5-hole salt shaker instead of their usual salt shakers.

**Generation of customer demand** (*n* = 3): Three businesses reported the generation of customer demand as a result of implementing the intervention. Their customers reported that they liked the fact that there were more healthier choices on the menu. One evaluation of an intervention [Award 41] reported that they were selling more water and diet drinks now that these are more prominently displayed in their outlet.

## Discussion

### Summary of findings

To our knowledge this is the first systematic mapping and evidence synthesis of interventions to promote healthier ready-to-eat-food sold by specific food outlets in England. We identified 75 interventions with information on content and delivery. Evaluations were conducted on 30 these 75 interventions. The majority (43 of 75) of interventions were awards, which tended to be aimed at a broad range of food outlets and target multiple nutrients for change. In contrast, non-award interventions tended to be aimed at independently owned foot outlets and target specific nutrients.

The majority of project teams who promoted the uptake of interventions by food outlets were local government workers, and most commonly they were environmental health officers. Funding for the projects was usually time-limited, and the delivery of interventions tended to be cost-neutral to the food outlets.

Food outlets were offered a range of support, including in some cases training and provision of new equipment. The most common practice targeted by interventions was adaptation of existing cooking practices. Adding ‘healthy meal’ options, smaller portion size options, menu labelling, and healthier choices on children’s menus, were also popular. There was some evidence to suggest that if interventions can be implemented there is a strong likelihood that changes to food outlet practices will be maintained.

Evaluations predominately focused on acceptability of interventions to business owners. Evaluation findings suggest that successful delivery and implementation of these interventions requires a substantial time commitment from the project team with key personal skills and knowledge. Businesses were more likely to engage with cost neutral interventions which were relatively easy to implement, and those which offered imperceptible changes to price, palatability and portion size. Some businesses did find difficulties in sourcing healthier ingredients at affordable prices.

### Strengths and limitations of methods

We used novel and systematic methods to search for relevant interventions and evaluations. By using these methods we identified over 100 relevant interventions. However, of course, we cannot be sure that we identified all relevant interventions. Building on the search methods used in this paper and that of Godin et al. [30], feasible and robust methods for applying systematic search strategies to identify web-based and desk-based information in the grey literature that are of relevance to public health are needed.

Our ability to draw conclusions was limited by the quality of reporting of information on intervention content and delivery available, and the limited scope and low methodological quality of evaluations. In nearly all cases, evaluation results were favourable about the intervention, but these findings need to be considered with some caution for two reasons. First, in all cases, evaluations were conducted to inform service delivery rather than as formal research. As such, evaluations were fit for practice, but were limited in scope and of low methodological quality for research purposes. Second, in most cases, evaluations had been conducted by project teams who were also responsible for promoting the uptake of the intervention by food outlet proprietors and their staff, and hence at risk of bias [31].

### Interpretation of findings

The rich findings of this review provide information about the scope, specific features, and delivery of existing interventions in England. In addition, the findings provide useful information about aspects of the feasibility and process of the interventions identified. However, the findings only provide clues as to the impact of these interventions on ready-to-eat-meals sold by specific food outlets, and how this might influence the dietary intake of customers and public health.

Comparing the range of practices targeted by the interventions identified in this review with interventions from other countries [32], it is clear that the interventions operating in England are limited. Specifically, the use of price reductions, personalised receipts, telemarketing and/or mandatory legislation used in other countries, were entirely absent here. Some of these approaches may be hard for local actors to implement particularly in independently owned food outlets in areas of deprivation.

In particular, very few interventions involved working upstream with food suppliers, generating customer demand, changing competition effects, or reducing portion sizes. All of these options, at least in theory [33–35], could be useful practices to target. Also, few of the interventions operated at a population level. Population level interventions have the advantage that they are often more effective and equitable than more individualistic interventions, although have not been popular with governments in the UK [36, 37].

### Implications for policy and practice

The fact that there is such a diversity of schemes in operation across England makes it difficult to compare their feasibility and impact, and this must be confusing for consumers, and contribute to their general lack of awareness and understanding of the schemes.

We recommend the rich source of information presented in this paper is captured, ideally by Public Health England (PHE), who then facilitate the sharing of good practice between project teams. Given the similar context in other countries, particularly Ireland, Scotland and Wales, we suggest these findings have currency beyond England. We also suggest that PHE assesses the transferability of findings presented in this paper (for example, between chain and independent food outlets, and between areas of low and high deprivation), and translate the available evidence within a useful resource (such as a toolkit) that delivers practical and pragmatic support to project teams who are responsible for promoting the uptake of interventions to food outlet proprietors.

### Implications for research

Our findings have identified two key findings for research.

First, we found few rigorous evaluations of interventions; the lack of robust evaluations of these sort of initiatives and the difficulty in conducting them (e.g. because of difficulty in undertaking nutritional analysis of food due to lack of standardised menus in independent food outlets) are particularly pertinent. More consideration should be given and efforts made to conduct rigorous evaluations of interventions to promote healthier ready-to-eat meals (to eat in, to take away, or to be delivered) sold by specific food outlets in England. We acknowledge that local authorities do not have the necessary resource for such evaluations. Researchers with specific expertise and knowledge in this area should engage and work in partnership with policy and practice staff that are developing, promoting and evaluating interventions at all levels, including the local level. Rigorous evaluations should include outcome as well as process analysis. Ideally, impacts on inequalities, and variations in effect by type of food outlet, and geographical areas should be captured.

Secondly, the feasibility of developing evidence based interventions in this area should be explored. We suggest a range of interventions should be tested, which target different behavioural change strategies at various system levels [38, 39]. Potentially promising approaches that deserve further attention include working upstream with suppliers; and working with communities to generate greater consumer demand for healthier alternatives. Other particularly common approaches that deserve further evaluation include ‘health by stealth’ approaches, reducing portion sizes, and changing the balance of healthy to less healthy options.

## Conclusions

This systematic mapping and evidence synthesis of interventions to promote healthier ready-to-eat-food sold by specific food outlets in England provides information to help inform the development, implementation and evaluation of interventions. The best available evidence suggests that food outlet proprietors are generally positive about implementing these interventions, particularly when they are cost neutral and use a health by stealth approach. Little robust evidence is available on the effectiveness of these approaches and further research is needed to generate this evidence. Opportunities for working upstream with suppliers, and in co-participation with consumers, when developing interventions should be explored.

## Additional files

Additional file 1: Process of categorisation of food outlets targeted by the interventions included in this review. (DOCX 16 kb)
Additional file 2: List of people contacted, and method(s) of contact, asking for information about interventions to promote healthier ready-to-eat meals (to eat in, take away, or delivered) sold by specific food outlets in England. (DOCX 25 kb)
Additional file 6: Summary of the content and delivery of interventions to promote healthier ready-to-eat meals (to eat in, take away, or delivered) sold by specifica food outlets in England (Tier 1, n = 75) (DOCX 38 kb)
Additional file 3: List (name and location) of interventions to promote healthier ready-to-eat meals (to eat in, take away, or delivered) sold by specific1 food outlets in England and identification and data sources (Tier 1, *n* = 75). (DOCX 17 kb)
Additional file 4: List (name and location) of interventions to promote healthier ready-to-eat meals (to eat in, take away, or delivered) sold by specific1 food outlets in England identified through searches but excluded for the reason of insufficient information. (DOCX 14 kb)
Additional file 5: Description of the content and delivery of interventions to promote healthier ready-to-eat meals (to eat in, take away, or delivered) sold by specific food outlets in England (Tier 1, *n* = 75). (DOCX 81 kb)
Additional file 7: Description of the design, methods and results of evaluations of interventions to promote healthier ready-to-eat meals (to eat in, take away, or delivered) sold by specific food outlets in England (Tier 2, *n* = 30). (DOCX 63 kb)

## Abbreviations

HCC: Healthy Catering Commitment
PHE: Public Health England
