# Supplementary material for: A description of interventions promoting healthier ready-to-eat meals (to eat in, to take away, or to be delivered) sold by specific food outlets in England: a systematic mapping and evidence synthesis
Source: BMC Public Health. 2017 Jan 19;17:93. doi: 10.1186/s12889-016-3980-2 (PMC5244522; doi:10.1186/s12889-016-3980-2)
Supplement: Additional file 1: — Process of categorisation of food outlets targeted by the interventions included in this review. (DOCX 16 kb) [file 12889_2016_3980_MOESM1_ESM.docx]

**Additional file 1: Process of categorisation of food outlets targeted by the interventions included in this review.**

| List of categories of food outlets developed by Lake et al [24, 25] deemed relevant for this review (food outlets could map onto more than one category):   1. **Baker-retail outlet** *(which are takeaway only)* 2. **Café/Coffee Shop** 3. **Hotel food outlets** *(which are eat-in only)* 4. **Fast Food outlet** 5. **Pizzeria** 6. **Pub/Bar** *(which are eat-in only)* 7. **Restaurant** 8. **Sandwich Shop** 9. **Takeaway food outlet** *(which are takeaway only, includes mobile food outlets and market stalls)*   Where an intervention targeted a specific type of food outlet, a note was made as to whether this type of food outlet offered customers meals to eat-in only, to takeaway and/or be delivered, or a combination of both. Note: many of the interventions included in this review targeted a range of food outlets.  Following this process, these nine categories were compressed into three categories:   1. Takeaway (only) eateries (to takeaway and/or be delivered) 2. Sit-in (only) eateries 3. Sit-in and takeaway (to takeaway and/or be delivered) eateries |
| --- |

24. Lake A, Burgoine T, Stamp E, Grieve R: The foodscape: classification and field validation of secondary data sources across urban/rural and socio-economic classifications in England. Int J Behav Nutr Phys Act 2012, 9(1):37.

25. Lake AA, Burgoine T, Greenhalgh F, Stamp E, Tyrrell R: The Foodscape: classification and field validation of secondary data sources. Health Place 2010, 16 (4):666-673.
