# Supplementary material for: A description of interventions promoting healthier ready-to-eat meals (to eat in, to take away, or to be delivered) sold by specific food outlets in England: a systematic mapping and evidence synthesis
Source: BMC Public Health. 2017 Jan 19;17:93. doi: 10.1186/s12889-016-3980-2 (PMC5244522; doi:10.1186/s12889-016-3980-2)
Supplement: Additional file 2: — List of people contacted, and method(s) of contact, asking for information about interventions to promote healthier ready-to-eat meals (to eat in, take away, or delivered) sold by specific food outlets in England. (DOCX 25 kb) [file 12889_2016_3980_MOESM2_ESM.docx]

### Additional file 2: List of people contacted, and method(s) of contact, asking for information about interventions to promote healthier ready-to-eat meals (to eat in, take away, or delivered) sold by specific food outlets in England.

## Local authorities in England: all 353 Local Authorities in England were contacted by email initially, and those that did not respond were then contacted by telephone, to obtain the personal email addresses of key personnel working in public and environmental health, and an email was sent to relevant contacts.

## Topic experts based in UK: an email was sent to 20 topic experts (all UK based) that were identified by the research team as having expertise in this area.

**Relevant health professionals and workers, using social media:** Information was requested via the social media Twitter and LinkedIn to British Dietetic Association (BDA) members only group, North East Obesogenic environment Network (NEOeN) members group, European Nutrition Leadership Programme members group, Dietetic Research Network, International Obesity Forum, Association for the Study of Obesity members group, members of the Society for Nutrition Education and Behaviour, and members of the Chartered Institute of Environmental Health.

**Relevant health professionals and workers, using various other communication methods:** Information was requested using short articles which were appeared in routine newsletters, magazines, bulletins and websites for members of the BDA, Association for Nutritionists (AFN), Nutrition Society, Association of Directors of Public Health and Network Health Group. In addition, requests for information were sent to appropriate email distributions lists; BDA, AFN, Nutrition Society, Contact Help Advice and Information Network, Self Employed Nutritionists Support and Enlightenment, and the British Sociological Society Food Study special interest group.
