# Supplementary material for: A description of interventions promoting healthier ready-to-eat meals (to eat in, to take away, or to be delivered) sold by specific food outlets in England: a systematic mapping and evidence synthesis
Source: BMC Public Health. 2017 Jan 19;17:93. doi: 10.1186/s12889-016-3980-2 (PMC5244522; doi:10.1186/s12889-016-3980-2)
Supplement: Additional file 6: — Summary of the content and delivery of interventions to promote healthier ready-to-eat meals (to eat in, take away, or delivered) sold by specifica food outlets in England (Tier 1, n = 75) (DOCX 38 kb) [file 12889_2016_3980_MOESM6_ESM.docx]

**Additional file 6: Summary of the content and delivery of interventions to promote healthier ready-to-eat meals (to eat in, take away, or delivered) sold by specific^1^ food outlets in England** (Tier 1, n=75).

| **Project name (reference number)** | **Type of food outlet targeted by the intervention^2^, and notes^3^** | **Description of support^4^ provided by the project team to the food outlets proprietors and their staff** | | | | | | | | | | **Description of the practices^5^ that food outlets were asked to change as part of the intervention** | | | | | | | |
| --- | --- | --- | --- | --- | --- | --- | --- | --- | --- | --- | --- | --- | --- | --- | --- | --- | --- | --- | --- |
|  |  | **Skills training** | **Personalised support/feedback** | **Nutritional analysis** | **Equipment provision** | **Information provision** | **Work with suppliers** | **Assessment** | **Accreditation** | **Re-assessment** | **Creation of customer demand** | **Menu labelling** | **Marketing/ promotion** | **Customer information** | **Reducing portion size** | **Removing options** | **Adding options** | **Change cooking practices** | **Suitable options for children** |
| Heart of Derbyshire (healthier catering award)  (Award 1) | Takeaways and Sit in eateries (1, 2 and 3) |  |  |  |  |  |  | ✓ | ✓ | ✓ |  |  |  |  |  |  |  |  |  |
| Rochdale Borough Council’s Healthier Chips  (Award 2) | Takeaway eateries (1)  Near: specifically outlets near schools | ✓ |  |  | ✓ |  |  | ✓ | ✓ |  |  |  |  |  |  | ✓ |  | ✓ |  |
| Essex Healthy Eating Award Scheme  (Award 3) | Takeaways and Sit in eateries (1, 2 and 3) |  |  |  |  | ✓ |  | ✓ | ✓ |  |  | ✓ | ✓ |  |  |  | ✓ | ✓ | ✓ |
| Heart of Newcastle Award  (Award 4) | Takeaways and Sit in eateries (1, 2 and 3) |  |  |  |  | ✓ |  | ✓ | ✓ |  |  | ✓ |  |  |  |  | ✓ |  |  |
| Food for Life Catering Mark, Soil Association, UK wide  (Award 5) | Takeaways and Sit in eateries (1, 2 and 3) |  |  |  |  |  |  | ✓ | ✓ |  |  | ? |  |  |  |  | ? | ? |  |
| The Cornwall Healthier Eating and Food Safety (CHEFS) Award  (Award 6) | Takeaways and Sit in eateries (1, 2 and 3) |  |  |  |  |  |  | ✓ | ✓ |  |  | ? |  |  |  |  | ✓ | ? |  |
| Healthier Catering Commitment, Cambridgeshire  (Award 7) | Takeaway eateries (1)  Notes: included outlets near schools, areas of high deprivation |  |  |  |  |  |  | ✓ | ✓ |  |  |  |  |  |  |  |  | ? |  |
| Good Food Bradford Project  (Award 8) | Takeaway eateries (1) | ✓ |  |  |  | ✓ |  | ✓ | ✓ |  |  | ✓ | ✓ |  |  |  | ✓ | ✓ |  |
| Food4Health: Healthy eating awards, Middlesbrough  (Award 9) | Takeaways and Sit in eateries (1, 2 and 3) |  | ✓ | ✓ |  |  |  | ✓ | ✓ |  |  | ? | ✓ |  | ? |  | ✓ | ✓ | ✓ |
| Kirklees Healthy Choice Award  (Award 10) | Takeaways and Sit in eateries (1, 2 and 3) |  |  |  |  |  |  | ✓ | ✓ |  |  | ✓ |  |  |  |  | ✓ |  |  |
| Dudley Food for Health Award  (Award 11) | Takeaways and Sit in eateries (1, 2 and 3) | ✓ |  |  |  | ✓ |  | ✓ | ✓ |  |  |  |  |  |  |  |  |  |  |
| Healthy Eating Award, Tonbridge and Malling  (Award 12) | Takeaways and Sit in eateries (1, 2 and 3) |  | ✓ |  |  | ✓ |  | ✓ | ✓ |  |  |  |  |  |  |  |  |  |  |
| Healthy Catering Award, Blackpool  (Award 13) | Sit in eateries and Takeaways (1,2 and 3) |  |  |  |  |  |  | ✓ | ✓ |  |  |  |  |  |  | ? | ? | ? |  |
| ‘Eat Out Eat Well' scheme, Surrey, Bath & North East Somerset, Crawley, West Berkshire, Wokingham and Medway  (Award 14) | Takeaways and Sit in eateries (1, 2 and 3) | ✓ |  |  |  |  |  | ✓ | ✓ |  |  |  |  |  |  |  | ✓ | ✓ |  |
| Recipe4Health, Lancashire  (Award 15) | Takeaways and Sit in eateries (1, 2 and 3) |  |  | ✓ |  | ✓ |  | ✓ | ✓ |  |  |  |  |  |  |  |  |  | ✓ |
| Central England Trading Association Truckers Tucker  (Award 16) | Takeaways and Sit in eateries (1, 2 and 3) |  |  |  |  | ✓ |  | ✓ | ✓ |  |  |  |  |  |  |  |  |  |  |
| Brighton and Hove Healthy Catering Award  (Award 17) | Takeaways and Sit in eateries (1, 2 and 3) |  |  |  |  |  |  | ✓ | ✓ |  |  | ✓ | ✓ |  |  | ? | ✓ | ✓ |  |
| London Healthy Catering Commitment (Eat Well Croydon)  (Award 18) | Takeaway eateries (1)  Notes: outlets in disadvantaged areas |  | ✓ |  |  |  |  | ✓ | ✓ |  |  |  | ✓ | ✓ |  | ✓ | ✓ | ✓ |  |
| Nottinghamshire County Council fast food outlet ‘merit scheme’  (Award 19) | Takeaway eateries (1) |  | ✓ |  |  | ✓ |  | ✓ | ✓ |  |  |  |  |  |  |  |  |  |  |
| Tower Hamlets Healthy Towns/Healthy Food Award/Food for Health  (Award 20) | Takeaways and Sit in eateries (1, 2 and 3) | ✓ |  |  |  |  |  | ✓ | ✓ |  |  |  |  |  | ✓ |  | ✓ | ✓ |  |
| Healthier Options Norfolk Award (HONOR),  (Award 21) | Takeaways and Sit in eateries (1, 2 and 3) |  |  |  |  |  | ✓ | ✓ | ✓ |  |  |  | ✓ |  | ✓ |  | ✓ | ✓ | ✓ |
| Tunbridge Wells Healthy Choices Award  (Award 22) | Takeaways and Sit in eateries (1, 2 and 3) |  | ✓ |  |  | ✓ | ✓ | ✓ | ✓ |  |  |  |  |  |  |  | ✓ | ✓ |  |
| Heartbeat award, Warwickshire  (Award 23) | Takeaways and Sit in eateries (1, 2 and 3) |  |  |  |  |  |  | ✓ | ✓ |  |  | ✓ |  |  | ✓ |  | ✓ | ? |  |
| St Helens Healthier Chip project (Chip fryer Award)  (Award 24) | Takeaway eateries (1) | ✓ |  |  |  | ✓ |  | ✓ | ✓ |  |  |  | ✓ |  |  |  |  | ✓ |  |
| Bristol Better Sandwiches project  (Award 25) | Takeaway eateries (1)  Notes: independent outlets only |  |  |  |  | ✓ |  |  | ✓ |  |  |  | ✓ |  |  | ✓ | ✓ | ✓ |  |
| Heartbeat Award (Health Education Authority), England-wide  (Award 26) [40, 41] | Takeaways and Sit in eateries (1, 2 and 3)  Notes: intervention aimed at lower SES groups |  | ✓ |  |  |  |  | ✓ | ✓ |  |  | ✓ | ✓ | ✓ |  | ? | ? | ? |  |
| Eat Well Award, Undisclosed PCT in the North West  (Award 27) [42] | Takeaways and Sit in eateries (1, 2 and 3)  Notes: outlets in disadvantaged areas |  |  |  |  |  |  | ✓ | ✓ |  |  | ? |  |  |  |  | ? | ? |  |
| Shropshire healthy eating award  (Award 28) | Takeaways and Sit in eateries (1, 2 and 3) |  | ✓ |  |  | ✓ |  | ✓ | ✓ |  |  |  | ✓ |  |  |  | ✓ | ✓ | ✓ |
| Healthy Business Award, Ashton, Leigh, Wigan  (Award 29) | Takeaways and Sit in eateries (1, 2 and 3)  Notes: included outlets in deprived areas |  |  |  |  |  |  | ✓ | ✓ |  |  | ✓ |  |  |  |  | ✓ | ✓ |  |
| Healthier Options Food Awards, Newham  (Award 30) | Takeaways and Sit in eateries (1, 2 and 3) | ✓ | ✓ |  |  |  |  | ? | ✓ |  |  |  |  |  |  | ? | ? | ? |  |
| Golden Apple Healthy Eating Award, Hartlepool  (Award 31) | Takeaways and Sit in eateries (1, 2 and 3) |  |  |  |  | ✓ |  | ✓ | ✓ |  |  | ✓ | ✓ |  |  |  | ✓ | ✓ |  |
| Greater Manchester Healthier Catering Award  (Award 32) | Takeaways and Sit in eateries (1, 2 and 3) |  |  |  |  |  |  | ✓ | ✓ |  |  |  | ✓ |  |  |  | ✓ | ? |  |
| Wakefield Eatwell award,  (Award 33) | Takeaways and Sit in eateries (1, 2 and 3) |  | ✓ |  |  |  |  |  | ✓ |  |  |  | ✓ |  |  |  | ? | ? |  |
| London Healthier Catering Commitment (overall),  (Award 34) (HCC)[26] | Takeaways and Sit in eateries (1, 2 and 3)  notes: included outlets in deprived areas |  |  |  |  |  |  | ✓ | ✓ |  |  |  |  |  |  |  | ✓ | ✓ | ✓ |
| London Healthy Catering Commitment, Lambeth  (Award 35) (HCC) | Takeaways and Sit in eateries (1, 2 and 3) |  | ✓ | ✓ |  |  |  | ✓ | ✓ |  |  |  |  |  |  |  | ✓ | ✓ | ✓ |
| London Healthy Catering Commitment, Harrow  (Award 36) (HCC) | Takeaway eateries (1) | ✓ |  |  |  |  |  | ✓ | ✓ |  |  |  |  |  |  |  | ✓ | ✓ | ✓ |
| London Healthy Catering Commitment, Barnet  (Award 37) (HCC) | Takeaways and Sit in eateries (1, 2 and 3) |  |  |  |  |  |  | ✓ | ✓ |  |  |  |  |  |  |  | ✓ | ✓ | ✓ |
| London Healthy Catering Commitment, Barking and Dagenham  (Award 38) (HCC) | Takeaway eateries (1) |  |  |  |  |  |  | ✓ | ✓ |  |  |  |  |  |  |  | ✓ | ✓ | ✓ |
| London Healthy Catering Commitment, Lewisham  (Award 39)  (HCC) | Takeaways and Sit in eateries (1, 2 and 3)  Notes: Outlets near schools and/or in most deprived wards, serving fried fish or chicken | ✓ |  |  | ✓ |  |  | ✓ | ✓ |  |  |  |  |  |  |  | ✓ | ✓ | ✓ |
| London Healthier Catering Commitment, Hammersmith and Fulham,  Kensington and Chelsea and Westminster  (Award 40) (HCC) | Takeaways and Sit in eateries (1, 2 and 3)  Notes: Outlets in affluent and deprived areas |  | ✓ |  |  |  |  | ✓ | ✓ |  |  |  |  |  |  |  | ✓ | ✓ | ✓ |
| London Healthy Catering Commitment, Sutton and Merton (incorporated in Sutton and Merton Responsibility Deal)  (Award 41) (HCC) | Takeaways and Sit in eateries (1, 2 and 3)  Notes: independent outlets |  |  |  |  |  |  | ✓ | ✓ |  |  |  |  |  |  |  | ✓ | ✓ | ✓ |
| London Healthy Catering Commitment, London Borough of Richmond (Whitton & Heathfield)  (Award 42) (HCC) | Takeaways and Sit in eateries (1, 2 and 3)  Notes: independent outlets |  |  |  |  |  |  | ✓ | ✓ |  |  |  | ✓ |  |  |  | ✓ | ✓ | ✓ |
| London Healthy Catering Commitment, London Borough of Richmond (Ham, Sheen and Twickenham)  (Award 43) (HCC) | Takeaways and Sit in eateries (1, 2 and 3)  Notes: outlets near schools |  |  |  |  |  |  | ✓ | ✓ |  |  |  | ✓ |  |  |  | ✓ | ✓ | ✓ |
| Healthy Fast Food Network, London  (Non-award 1) | Takeaway eateries (1)  Notes: Outlets near schools |  | ✓ | ✓ |  |  |  | ✓ | NA |  |  |  |  |  |  | ? | ? | ? |  |
| Torbay Healthy catering inserts  (Non-award 2) | Takeaways and Sit in eateries (1, 2 and 3) |  |  |  |  | ✓ |  |  | NA |  |  |  |  |  |  |  |  |  |  |
| Stoke-on-Trent takeaways near schools project  (Non-award 3) | Takeaway eateries (1)  Notes: Outlets near schools |  | ✓ |  |  |  |  |  | NA |  |  |  |  |  |  |  |  | ✓ | ✓ |
| Takeaway project targeting frying practice to reduce fat and calorie intake, Nottingham  (Non-award 4) | Takeaway eateries (1)  Notes: independent outlets, areas with high deprivation |  | ✓ |  |  |  |  |  | NA |  |  |  |  |  |  |  |  | ✓ |  |
| Sheffield takeaway project  (Non-award 5) | Takeaway eateries (1)  Notes: independent outlets |  | ✓ |  |  | ✓ |  | ✓ | NA |  | ✓ |  |  | ✓ |  |  |  |  |  |
| Shropshire Takeaway project  (Non-award 6) | Takeaway eateries (1)  Notes: outlets near schools |  | ✓ |  |  | ✓ |  | ✓ | NA | ✓ |  | ✓ |  |  | ? |  | ? | ✓ |  |
| Out to Lunch rating system, UK wide  (Non-award 7) | Sit in eateries (2)  Notes: includes chain outlets |  | ✓ |  |  | ✓ |  | ✓ | NA |  |  |  |  | ✓ |  | ✓ | ✓ | ✓ | ✓ |
| Researches project "Supporting interventions for healthier catering: tools and resources for SMEs in the independent fast food sector", London  (Non-award 8) | Takeaways and Sit in eateries (1, 2 and 3)  Notes: independent outlets, deprived areas |  |  |  |  | ✓ | ✓ |  | NA |  |  |  |  |  |  | ✓ | ✓ | ✓ |  |
| Eatright Liverpool  (Non-award 9) | Takeaways and Sit in eateries (1, 2 and 3) | ✓ | ✓ | ✓ | ✓ | ✓ |  |  | NA |  |  |  | ✓ |  |  |  |  | ✓ |  |
| Knowsley Healthy Eating project  (Non-award 10) | Takeaway eateries (1) | ✓ | ✓ | ✓ |  |  |  |  | NA | ✓ |  |  |  |  |  | ? | ? | ? |  |
| Stoke-on-Trent Asian takeaway project  (Non-award 11) | Takeaway eateries (1) |  |  |  |  |  |  |  | NA |  |  | ✓ |  |  |  |  |  | ✓ |  |
| Café Vibe project at Beverley Leisure Centre, East Riding of Yorkshire  (Non-award 12) | Sit in eateries (2) | ✓ | ✓ |  |  |  |  |  | NA |  |  | ✓ |  |  |  |  |  | ✓ |  |
| Food business training project in combination with a healthy eating project, Luton  (Non-award 13) | Takeaways and Sit in eateries (1, 2 and 3) |  |  |  |  | ✓ |  | ✓ | NA | ✓ |  |  |  |  |  |  |  | ✓ |  |
| Take-away masterclasses, Kirklees  (Non-award 14) | Takeaways and Sit in eateries (1, 2 and 3) | ✓ |  |  |  |  |  |  | NA |  |  | ? | ✓ |  |  |  | ✓ | ✓ |  |
| Worcestershire Truckers Tucker  (Non-award 15) | Takeaways and Sit in eateries (1, 2 and 3) |  | ✓ | ✓ | ✓ | ✓ |  |  | NA |  |  |  |  |  |  | ? | ? | ? |  |
| Central England Trading Association Truckers Tucker  (Non-award 16) | Takeaways and Sit in eateries (1, 2 and 3) | ✓ | ✓ |  |  | ✓ |  |  | NA |  |  |  | ✓ |  | ✓ | ✓ | ✓ | ✓ |  |
| Shropshire Eat Well live Longer - on the road  (Non-award 17) | Takeaways and Sit in eateries (1, 2 and 3)  Notes: outlets in areas of social deprivation |  | ✓ |  | ✓ |  |  | ✓ | NA | ✓ |  |  | ✓ |  |  |  | ? | ? |  |
| Warwickshire Truckers Tucker  (Non-award 18) | Takeaways and Sit in eateries (1, 2 and 3) |  | ✓ |  | ✓ | ✓ |  |  | NA |  |  |  |  |  | ✓ |  | ✓ | ✓ |  |
| Lincolnshire eat in, eat out, eat healthy  (Non-award 19) | Takeaways and Sit in eateries (1, 2 and 3) |  | ✓ |  |  | ✓ | ✓ |  | NA |  | ✓ | ✓ | ✓ | ✓ | ✓ |  | ✓ | ✓ |  |
| Lighting the Beacons project - healthier takeaways  (Non-award 20) | Takeaways and Sit in eateries (1, 2 and 3) |  |  |  |  |  | ✓ |  | NA |  |  |  |  |  |  |  |  |  |  |
| Healthier menu choices for children, South Somerset  (Non-award 21) | Takeaways and Sit in eateries (1, 2 and 3)  Notes: independent outlets |  | ✓ | ✓ |  |  |  |  | NA |  |  |  | ✓ |  |  | ? | ? | ✓ | ✓ |
| East Wirral Takeaway for change  (Non-award 22) | Takeaway eateries (1) |  |  |  | ✓ |  |  |  | NA |  |  | ✓ |  | ✓ |  |  | ✓ | ✓ |  |
| Box chicken, London  (Non-award 23) | Takeaway eateries (1)  Notes: outlets near schools, particularly in low income areas |  |  |  |  |  |  |  | NA |  |  |  | ✓ |  |  |  | ✓ |  |  |
| Enfield healthier takeaways project  (Non-award 24) | Takeaway eateries (1) |  | ✓ | ✓ | ✓ |  |  |  | NA | ✓ |  |  |  |  |  |  | ✓ | ✓ |  |
| Stoke-on-Trent Chip shop project  (Non-award 25) | Takeaway eateries (1) |  |  |  | ✓ | ✓ |  |  | NA |  |  |  | ✓ |  |  |  |  | ✓ |  |
| Shake Less Salt campaign, Norfolk  (Non-award 26) | Takeaway eateries (1) |  |  |  | ✓ |  |  |  | NA |  |  |  | ✓ | ✓ |  |  |  |  |  |
| Gateshead Salt Shakers  (Non-award 27) | Takeaway eateries (1) |  |  |  | ✓ | ✓ |  |  | NA |  |  |  | ? | ✓ |  |  |  |  |  |
| Sandwich project, Exeter  (Non-award 28) | Takeaway eateries (1) |  | ✓ |  |  | ✓ |  | ✓ | NA |  |  |  |  |  |  |  |  | ✓ |  |
| Sandwich project, Buckinghamshire  (Non-award 29) | Takeaway eateries (1) | ✓ |  |  |  | ✓ | ✓ | ✓ | NA |  |  |  | ✓ | ✓ |  | ✓ |  | ✓ |  |
| My Choice, London  (Non-award 30) | Takeaways and Sit in eateries (1, 2 and 3)  Notes: outlets in a deprived area |  |  |  |  | ✓ |  |  | NA |  |  | ✓ |  |  | ? |  | ? | ? |  |
| FSA project - calorie information at the point of choice in catering outlets, UK wide  (Non-award 31) | Takeaways and Sit in eateries (1, 2 and 3) |  |  |  |  | ✓ |  |  | NA |  |  | ✓ |  |  |  |  |  |  |  |
| Stoke-on-Trent Truckers Tucker  (Non-award 32) | Takeaways and Sit in eateries (1, 2 and 3) |  |  |  |  | ✓ |  |  | NA |  |  |  | ✓ |  |  |  |  | ✓ |  |

✓ = support or practice included in the intervention; ? = unclear if support or practice included in intervention but some implication that it may be; NA = not applicable

^1^The specific food outlets included were those that, as their main business, sold ready-to-eat meals and were openly accessible to the general public.

^2^Food outlets targeted by the intervention were mapped (see Additional file 1 for detail of process) onto one of three categories:

1. Takeaway eateries (takeaways)
2. Sit-in eateries
3. Food outlets that included options to takeaway or sit-in

^3^Information on whether the intervention included chain and/or independent outlets, and/or had a particular focus on low SES groups or outlets near schools, where reported

^4^A specific action or set of actions undertaken by the project team that aimed to engage and enable the food outlets with change. Operationalised as the description of any behaviours or cluster of behaviours enacted by the project team to support food outlets with change

^5^A specific action or set of actions undertaken by the food outlets that aimed to promote and/or offer healthier ready-to-eat meals. Operationalised as the description of the target behaviour or cluster of behaviours enacted by the food outlets to promote and/or offer healthier ready-to-eat meals.
