# Supplementary material for: A description of interventions promoting healthier ready-to-eat meals (to eat in, to take away, or to be delivered) sold by specific food outlets in England: a systematic mapping and evidence synthesis
Source: BMC Public Health. 2017 Jan 19;17:93. doi: 10.1186/s12889-016-3980-2 (PMC5244522; doi:10.1186/s12889-016-3980-2)
Supplement: Additional file 3: — List (name and location) of interventions to promote healthier ready-to-eat meals (to eat in, take away, or delivered) sold by specific1 food outlets in England and identification and data sources (Tier 1, n = 75). (DOCX 17 kb) [file 12889_2016_3980_MOESM3_ESM.docx]

**Additional file 3: List (name and location) of interventions to promote healthier ready-to-eat meals (to eat in, take away, or delivered) sold by specific^1^ food outlets in England and identification and data sources** (Tier 1, n=75).

| **Project reference** | **Name of intervention and location** | **Contact organisation** | **How identified** | **Data sources** |
| --- | --- | --- | --- | --- |
| Award 1 | Heart of Derbyshire (healthier catering award), Derbyshire | Derbyshire County Council | Local Authority (LA) contact (x2) and topic expert | Personal communication (email) |
| Award 2 | Rochdale Borough Council’s Healthier Chips, Rochdale | Rochdale Borough Council | LA contact, topic expert, health workers (via social media) (x3) | Personal communication (email) and website:  <http://www.rochdale.gov.uk/campaigns/healthy_chips_scheme.aspx> (accessed 11/02/2014) |
| Award 3 | Essex Healthy Eating Award Scheme | Healthy Life Essex | Health workers (via other methods) and topic expert | Personal communication (email) and website:  <http://www.healthylifeessex.co.uk/HealthyEating/HealthyEatingAward(about).html> (accessed 11/02/2014) |
| Award 4 | Heart of Newcastle Award, Newcastle | Newcastle City Council | LA contact | Personal communication (email) |
| Award 5 | Food for Life Catering Mark, Soil Association, UK wide | Soil Association | Topic expert | Personal communication (email) and website:  <http://www.sacert.org/catering/whatisthecateringmark>  (accessed 18/03/2014) |
| Award 6 | The Cornwall Healthier Eating and Food Safety (CHEFS) Award, Cornwall | Cornwall Council | Topic expert and Google searches (x2) | Personal communication (email; internal documents shared) and website:  <http://www.cornwall.gov.uk/health-and-social-care/food-safety/chefs-award/?page=17480>  (accessed 10/02/14) |
| Award 7 | Healthier Catering Commitment, Cambridgeshire | Cambridgeshire County Council | LA contact | Personal communication (email; internal documents shared) |
| Award 8 | Good Food Bradford Project, Bradford | Bradford District Metropolitan Council | Topic expert, health workers (via social media) and Google searches | Personal communication (email; internal documents shared) |
| Award 9 | Food4Health: Healthy eating awards, Middlesbrough | Middlesbrough Council | LA contact | Personal communication (email) |
| Award 10 | Kirklees Healthy Choice Award, Kirklees | Kirklees Council | Topic expert and website searches | Personal communication (email; internal documents shared) |
| Award 11 | Dudley Food for Health Award, Dudley | Dudley Metropolitan Borough Council | LA contact | Personal communication (email) |
| Award 12 | Healthy Eating Award, Tonbridge and Malling | Tonbridge and Malling Borough Council | Topic expert | Website: <http://www.tmbc.gov.uk/services/business/health-and-safety/food-business-health-promotion>  (accessed 27/03/2014) |
| Award 13 | Healthy Catering Award, Blackpool | Blackpool Council | LA contact | Personal communication (email; internal documents shared) |
| Award 14 | ‘Eat Out Eat Well' scheme, Surrey, Bath & North East Somerset, Crawley, West Berkshire, Wokingham and Medway | Surrey County Council and Bath and North East Somerset Council | LA contact (x2) and topic expert | Personal communication (email; internal documents shared) |
| Award 15 | Recipe4Health, Lancashire | Blackburn with Darwen Borough Council | LA contact | Personal communication (email; internal documents shared) |
| Award 16 | Central England Trading Association Truckers Tucker, Central England | Walsall Council | LA contact | Personal communication (email) |
| Award 17 | Brighton and Hove Healthy Catering Award, Brighton and Hove | Brighton and Hove City Council | Topic expert | Personal communication (email) |
| Award 18 | London Healthy Catering Commitment (Eat Well Croydon), Croydon | Croydon Council | Google searches | Personal communication (email; internal documents shared) |
| Award 19 | Nottinghamshire County Council fast food outlet ‘merit scheme’, Nottinghamshire | Nottinghamshire County Council | LA contact (x2) | Personal communication (email) |
| Award 20 | Tower Hamlets Healthy Towns/Healthy Food Award/Food for Health, Tower Hamlets | Tower Hamlets Council | Topic expert, health workers (via other methods) and Google searches (x2) | Personal communication (email; internal reports shared), website:  <http://www.towerhamlets.gov.uk/lgsl/401-450/409_healthy_food_awards.aspx>  (accessed 11/02/2014)  and report: ‘Active lifestyles and healthy eating: views from Tower Hamlets communities’ available at <http://shortwork.org.uk/files/report_web.pdf> (accessed 10/02/2014) |
| Award 21 | Healthier Options Norfolk Award (HONOR), Norfolk | Norwich City Council | Topic expert | Personal communication (email) and website: <http://www.norwich.gov.uk/Environment/EnvironmentalHealth/FoodSafety/pages/HealthierOptionsNorfolk.aspx> (accessed 14/04/2014) |
| Award 22 | Tunbridge Wells Healthy Choices Award, Tunbridge Wells | Tunbridge Wells Borough Council | LA contact | Personal communication (email) and website:  <http://www.tunbridgewells.gov.uk/business/environmental-health/food-hygiene/healthy-choices-award> (accessed 14/03/2014) |
| Award 23 | Heartbeat award, Warwickshire | North Warwickshire Borough Council | LA contact and health workers (via other methods) | Personal communication (email; internal documents shared) |
| Award 24 | St Helens Healthier Chip project (Chip fryer Award), St Helens, Merseyside | St. Helens Council | LA contact (x2) | Personal communication (email; internal documents shared) and website: [www.sthelens.gov.uk/chipaward](http://www.sthelens.gov.uk/chipaward) (accessed 13/03/2014) |
| Award 25 | Bristol Better Sandwiches project, Bristol | Bristol City Council | LA contact | Personal communication (email; internal documents shared) |
| Award 26 | Heartbeat Award (Health Education Authority), England-wide | Unclear | Topic expert | Peer-reviewed journal articles: Warm et al 1997 and Holdsworth et al 1997 |
| Award 27 | Eat Well Award, Undisclosed PCT in the North West | Not reported | Bibliographic databases and grey literature databases | Peer-reviewed journal article: Hanratty et al 2012 |
| Award 28 | Shropshire healthy eating award, Shropshire | Shropshire Council | LA contact | Personal communication (email; internal documents shared) |
| Award 29 | Healthy Business Award, Ashton, Leigh, Wigan | Wigan Council | LA contact, topic expert (x2), website searches and Google searches | Personal communication (email; internal documents shared) |
| Award 30 | Healthier Options Food Awards, Newham | Well London | Google searches | Personal communication (email) and website: [http://www.welllondon.org.uk/381/healthier -options-food-awards-buywell.html](http://www.welllondon.org.uk/381/healthier%20-options-food-awards-buywell.html) (accessed 10/02/2014) |
| Award 31 | Golden Apple Healthy Eating Award, Hartlepool | Hartlepool Borough Council | Health workers (via other methods) | Personal communication (email; internal documents shared) |
| Award 32 | Greater Manchester Healthier Catering Award, Greater Manchester | Salford City Council | LA contact and health workers (via social media) | Personal communication (email) and website: [http://www.healthiercatering.co.uk/about -the-scheme.aspx](http://www.healthiercatering.co.uk/about%20-the-scheme.aspx) (accessed 11/02/2014) |
| Award 33 | Wakefield Eatwell award, Wakefield | Wakefield Council | Topic expert and Google searches | Website: <http://www.wakefield.gov.uk/Environment/EnvironmentalHealth/FoodSafety/eatwell/apply_new.htm> (accessed 06/07/2014) |
| Award 34 | London Healthier Catering Commitment (overall), London | Chartered Institute for Environmental Health | LA contact, topic expert, health workers (via other methods) and Google searches | Personal communication (email; internal documents shared), peer-reviewed journal article: Bagwall 2014, and website: <http://www.cieh.org/healthier-catering-commitment.html> (accessed 11/02/2014) |
| Award 35 | London Healthy Catering Commitment, Lambeth | Lambeth Council | LA contact | Personal communication (email) |
| Award 36 | London Healthy Catering Commitment, Harrow | Harrow Council | LA contact and Google searches | Personal communication (email; internal documents shared) |
| Award 37 | London Healthy Catering Commitment, Barnet | Barnet Council | Google searches | Website: <http://www.barnet.gov.uk/info/930367/healthier_catering_award/1006/healthier_catering_award> (accessed 23/03/2014) |
| Award 38 | London Healthy Catering Commitment, Barking and Dagenham | London Borough of Barking and Dagenham Council | LA contact, grey literature databases and Google searches | Personal communication (email) and website: <http://www.lbbd.gov.uk/News/PressReleases/Pages/Healthyoptions.aspx> (accessed 27/03/2014) |
| Award 39 | London Healthy Catering Commitment, Lewisham | Lewisham Council | Google searches | Personal communication (email) and report: ‘Progress on the implementation of the Healthier Catering Commitment scheme in Lewisham’ available at <http://www.google.co.uk/url?sa=t&rct=j&q=&esrc=s&source=web&cd=1&ved=0ahUKEwjatoOvivDLAhUD7BQKHVE2AN0QFggcMAA&url=http%3A%2F%2Fcouncilmeetings.lewisham.gov.uk%2Fdocuments%2Fs27003%2F07HealthierCatering050214.pdf&usg=AFQjCNE6AAb9DmWcYWToiHZhq4SQusKHxA> (accessed 31/03/2014) |
| Award 40 | London Healthier Catering Commitment, Hammersmith and Fulham,  Kensington and Chelsea and Westminster | Westminster City Council | LA contact (x2) and Google searches | Personal communication (email; internal documents shared) |
| Award 41 | London Healthy Catering Commitment, Sutton and Merton (incorporated in Sutton and Merton Responsibility Deal) | London Borough of Sutton Council | LA contact and Google searches | Personal communication (email; internal documents shared) |
| Award 42 | London Healthy Catering Commitment, London Borough of Richmond (Whitton & Heathfield) | London Borough of Richmond Council and Nutrinsight | Health workers (via other methods) and Google searches | Personal communication (email) and report available at <http://www.nutrinsight.co.uk/services/public-health-projects>  (accessed 14/03/2014) |
| Award 43 | London Healthy Catering Commitment, London Borough of Richmond (Ham, Sheen and Twickenham) | London Borough of Richmond Council and Nutrinsight | Health workers (via other methods) and Google searches | Personal communication (email) and report available at <http://www.nutrinsight.co.uk/services/public-health-projects> (accessed 14/03/2014) |
| Non-award 1 | Healthy Fast Food Network, London | We Are What We Do (http://wearewhatwedo.org/) | LA contact | Personal communication (email; internal documents shared) |
| Non-award 2 | Torbay Healthy catering inserts, Torbay | Torbay Council | LA contact | Personal communication (email) |
| Non-award 3 | Stoke-on-Trent takeaways near schools project, Stoke-on-Trent | Stoke-on-Trent City Council | LA contact | Personal communication (email) |
| Non-award 4 | Takeaway project targeting frying practice to reduce fat and calorie intake, Nottingham | Nottingham CityCare Partnership | Health workers (via other methods) | Personal communication (email) |
| Non-award 5 | Sheffield takeaway project, Sheffield | Sheffield City Council | LA contact | Personal communication (email) |
| Non-award 6 | Shropshire Takeaway project, Shropshire | Shropshire Council | LA contact | Personal communication (email; internal documents shared) |
| Non-award 7 | Out to Lunch rating system, UK wide | Soil Association | Topic expert | Personal communication (email) and website: <http://www.soilassociation.org/outtolunch> (accessed 03/04/2014) |
| Non-award 8 | Researches project "Supporting interventions for healthier catering: tools and resources for SMEs in the independent fast food sector", London | London Metropolitan University | Health workers (via other methods) | Personal communication (email; internal documents shared) |
| Non-award 9 | Eatright Liverpool, Liverpool | Liverpool City Council and Liverpool John Moores University | Topic expert (x2), Health workers (via other methods), website searches and Google searches | Personal communication (email) and website: <http://www.foodvision.cieh.org/pages/liverpool-eatright> (accessed 11/02/2014) |
| Non-award 10 | Knowsley Healthy Eating project, Knowsley | Knowsley Council | Health workers (via other methods) (x2) and Google searches | Personal communication (email) and confidential reports (used with permission) |
| Non-award 11 | Stoke-on-Trent Asian takeaway project, Stoke-on-Trent | Stoke-on-Trent City Council | LA contact | Personal communication (email) |
| Non-award 12 | Café Vibe project at Beverley Leisure Centre, East Riding of Yorkshire | East Riding of Yorkshire Council | LA contact | Personal communication (telephone) and website: <http://www.beverleyleisurecomplex.co.uk/beverley-leisure-complex/cafe-vibe-share/> (accessed 14/02/2014) |
| Non-award 13 | Food business training project in combination with a healthy eating project, Luton | Luton Borough Council | LA contact | Personal communication (email) |
| Non-award 14 | Take-away masterclasses, Kirklees | Kirklees Council | Topic expert and Google searches | Personal communication (email; internal documents shared) |
| Non-award 15 | Worcestershire Truckers Tucker, Worcestershire | Worcestershire Regulatory Services | Topic expert | Personal communication (email) |
| Non-award 16 | Central England Trading Association Truckers Tucker, Central England | Walsall Council | Google searches | Personal communication (email) |
| Non-award 17 | Shropshire Eat Well live Longer - on the road, Shropshire | Shropshire Council | LA contact | Personal communication (email; internal documents shared) |
| Non-award 18 | Warwickshire Truckers Tucker, Warwickshire | Warwickshire County Council | Google searches | Personal communication (email; internal documents shared) |
| Non-award 19 | Lincolnshire eat in, eat out, eat healthy, Lincolnshire | Lincolnshire County Council | Website searches and Google searches | Websites: <http://www.social-change.co.uk/healthier-takeaways/> (23/12/13)  <http://www.foodvision.cieh.org/> (accessed 13/01/2014) |
| Non-award 20 | Lighting the Beacons project - healthier takeaways, East Midlands | National Social Marketing Centre | Topic expert and Google searches | National Social Marketing Centre (2009) Lighting the Beacon – A profile of the ten Beacon Partnership Projects underway in England <http://www.thensmc.com/sites/default/files/9102_NSMC_beacon_v6.1_LR.pdf> (accessed 17/12/2013) |
| Non-award 21 | Healthier menu choices for children, South Somerset | South Somerset District Council | LA contact | Personal communication (email and telephone; internal documents shared) |
| Non-award 22 | East Wirral Takeaway for change, East Wirral | Wirral Council | Topic expert | Personal communication (email, internal documents shared) |
| Non-award 23 | Box chicken, London | We Are What We Do (http://wearewhatwedo.org/) | LA contact and Google searches (x2) | Personal communication (email; internal documents shared) and website: <http://createlondon.org/event/chicken-shop/> (accessed 24/04/2014) |
| Non-award 24 | Enfield healthier takeaways project, Enfield | Enfield Council | Google searches (x2) | Personal communication (email; internal documents shared) |
| Non-award 25 | Stoke-on-Trent Chip shop project, Stoke-on-Trent | Stoke-on-Trent City Council | LA contact (x2) and Google searches (x2) | Personal communication (email) and website: [https://responsibilitydeal.dh.gov.uk/salt -and-fat-reduction/](https://responsibilitydeal.dh.gov.uk/salt%20-and-fat-reduction/) (accessed 10/02/2014) |
| Non-award 26 | Shake Less Salt campaign, Norfolk | Norfolk County Council | Topic expert, Google searches and website searches | Personal communication (email; internal documents shared) and website: <http://www.foodvision.cieh.org/pages/norfolk-salt-shaker-project> (accessed 11/02/2014) |
| Non-award 27 | Gateshead Salt Shakers, Gateshead | Gateshead Council | Topic expert, Health workers (via social media) website searches and Google searches | Personal communication (email; internal documents shared) |
| Non-award 28 | Sandwich project, Exeter | Exeter City Council | LA contact | Personal communication (email; internal documents shared) |
| Non-award 29 | Sandwich project, Buckinghamshire | Buckinghamshire County Council (Project team can no longer be contacted) | LA contact and Google searches | Website: <http://www.foodvision.cieh.org/pages/healthy-sandwich-project> (accessed 10/20/2014) |
| Non-award 30 | My Choice, London | Nutrinsight | Health workers (via other methods) and Google searches | Personal communication (email) and report available at <http://www.nutrinsight.co.uk/services/public-health-projects> (accessed 14/03/2014) |
| Non-award 31 | FSA project - calorie information at the point of choice in catering outlets, UK wide | Food Standards Agency | Website searches | Report from <http://foodbase.org.uk/> (accessed 13/01/2014):  Clegg et al (2009) An Evaluation of Provision of Calorie Information by Catering Outlets: BMRB Social Researches – Qualitative. Food Standards Agency, London |
| Non-award 32 | Stoke-on-Trent Truckers Tucker, Stoke-on-Trent | Stoke-on-Trent City Council | Topic expert | Personal communication (email; internal documents shared) |
