# Supplementary material for: A description of interventions promoting healthier ready-to-eat meals (to eat in, to take away, or to be delivered) sold by specific food outlets in England: a systematic mapping and evidence synthesis
Source: BMC Public Health. 2017 Jan 19;17:93. doi: 10.1186/s12889-016-3980-2 (PMC5244522; doi:10.1186/s12889-016-3980-2)
Supplement: Additional file 4: — List (name and location) of interventions to promote healthier ready-to-eat meals (to eat in, take away, or delivered) sold by specific1 food outlets in England identified through searches but excluded for the reason of insufficient information. (DOCX 14 kb) [file 12889_2016_3980_MOESM4_ESM.docx]

**Additional file 4: List (name and location) of interventions to promote healthier ready-to-eat meals (to eat in, take away, or delivered) sold by specific^1^ food outlets in England identified through searches but excluded for the reason of insufficient information**

| **Name of intervention and location** | **How identified** |
| --- | --- |
| Ask Campaign – encouraging reduced salt use, Greater Manchester | Topic expert |
| Catering for Health, Bracknell Forest | LA contact and topic expert |
| Chester 'salt shaker' type project, Chester | Google searches |
| Croydon Heart Town programme, Croydon | Google searches |
| Cumbria healthy eating award for cafes, takeaways and caterers, Cumbria | LA contact |
| Eat Well Wirral, Wirral | Topic expert |
| Healthier Options Award, Hull | LA contact and topic expert |
| Healthy Chinese takeaway project, Unclear | Google searches |
| Heartbeat Award, Buckinghamshire | LA contact |
| London Healthy Catering Commitment – Commit to Healthy Catering, Camden | Google searches |
| London Healthy Catering Commitment, Greenwich | LA contact, Health workers (via other methods) and Google searches |
| London Healthy Catering Commitment, Hackney | LA contact |
| London Healthy Catering Commitment, Haringey | LA contact |
| London Healthy Catering Commitment, Islington | Health workers (via other methods) |
| London Healthy Catering Commitment, Southwark | Google searches |
| London Healthy Catering Commitment, Waltham Forest | LA contact and Google searches |
| Method on healthy eating to be included within SFBB packs, Exeter | LA contact |
| Northamptonshire Heartbeat Award, Northamptonshire | Topic expert |
| Responsibility deal and menu labelling pledge, Buckinghamshire | LA contact |
| Department of Health pilot project - healthier catering advice for Indian/South Asian restaurants and takeaways, Royal Borough of Kensington and Chelsea | LA contact |
| Tips on chips project, Royal Borough of Kensington and Chelsea | LA contact |
| SALT campaign, Buckinghamshire | LA contact |
| Sampling takeaway meals for levels of salt & fat, Hartlepool | LA contact |
| Sefton fryers project (Heart of Mersey), Sefton | Google searches |
| South Lakeland District Council Healthier Menus Award, South Lakeland | Topic expert |
| Torbay Eat Wise Award scheme, Torbay | LA contact |
| Worcestershire Heartbeat style award, Worcestershire | Topic expert |
| Working with catering establishments to offer healthy food alternatives within their menus and improve the provision of healthier meals, Dorset | LA contact |

^1^The specific food outlets included were those that, as their main business, sold ready-to-eat meals and were openly accessible to the general public.
