# Supplementary material for: A description of interventions promoting healthier ready-to-eat meals (to eat in, to take away, or to be delivered) sold by specific food outlets in England: a systematic mapping and evidence synthesis
Source: BMC Public Health. 2017 Jan 19;17:93. doi: 10.1186/s12889-016-3980-2 (PMC5244522; doi:10.1186/s12889-016-3980-2)
Supplement: Additional file 5: — Description of the content and delivery of interventions to promote healthier ready-to-eat meals (to eat in, take away, or delivered) sold by specific food outlets in England (Tier 1, n = 75). (DOCX 81 kb) [file 12889_2016_3980_MOESM5_ESM.docx]

**Additional file 5: Description of the content and delivery of interventions to promote healthier ready-to-eat meals (to eat in, take away, or delivered) sold by specific^1^ food outlets in England** (Tier 1, n=75).

| **Type (award or non award), reference, status (as of January to March 2014) and dates of project** | **Aims** | **Intervention description** | **Details of intervention team, expertise and award accredited by** |
| --- | --- | --- | --- |
| Award 1  Planned  Start date June 2014  End date not determined | Not stated | *Award criteria:*  Award level depends upon the food hygiene rating, how many pledges (focusing on food content, food promotion, business environment) are made and adherence to them. | Local County, Borough and District Councils. Public Health and Trading Standards and Environmental Health Teams  Trading Standards will administer and accredit the scheme and help to promote it, and Environmental Health staff will visit the premises and carry out the audits.  No nutrition/ dietetic expertise planned as yet |
| Award 2  On-going  Initial project June 2012 – Feb 2014. Now extended until Feb 2015. | Encourage healthier frying practices in takeaways by reducing the amount of **saturated fat** absorbed by chips. | *Award criteria:*  Bronze: businesses must use cooking practice of banging the basket 6 times after removal from the fat; use straight chips; and not sell or give away scraps  Silver: Above plus focuses on the size of the chip, temperature cooked at and the quality of the oil  Gold: currently being revamped to focus on portion size and research being undertaken about the possibility of delivering other healthier options in addition to above  *Additional intervention:*  Initial training to takeaways on how to achieve the scheme levels and how to monitor their oil. Oil monitors given to each takeaway committed to the silver award. | Local Borough Council  Expertise unclear |
| Award 3  On-going  A trial of the scheme began in July 2007 | To promote commercial food outlets to offer **healthy food** or at least healthy options. Where business operators are willing to serve and promote healthy food then they will receive an award (a two tier scheme). The Gold and Silver awards are for commercial food outlets that have made the effort to offer healthy food, or at least healthy options (for details, see below). | *Award criteria:*  Silver: business is committed to the following conditions:  • Keeping the level of **fats** and oils to a minimum in the food served, in particular **saturated fat**;  • Keeping the level of **salt** to a minimum in the food served;  • Keeping the level of **sugar** to a minimum in the food served;  • Making **fruit and vegetables** clearly available;  • Making **starchy foods** the main part of most meals;  • Providing healthy and nutritious food for children in places where children are served;  • Making sure that at least one third of the food served is clearly described as meeting healthy choice requirements  Gold: In addition to above, commitment to:  • Making sure that at least 50% of the foods served are clearly described as ‘healthy eating’.  • Meeting a series of specific requirements (over 100)  • Having an appropriate sales promotion and marketing strategy which works alongside the general principles of the ‘Healthy Eating’ Award and supports healthier eating.  *Additional intervention:*  A guidance pack aimed at businesses is available. This includes money saving ideas, tips for healthy options for different times of day and practical tips. | Developed by the local Council’s Environmental Health Departments.  Quality assurance conducted through auditing and inspection by Environmental Health Officers |
| Award 4  On-going  2007 - ongoing | Promote and provide **healthy food** and drink choices in catering outlets | *Award criteria:*  Criteria based on availability of healthier food and drink options, appropriate signposting on menu  *Additional intervention:*  Advice for businesses to encourage take up of the Award | Local City Council Regulatory Services and Public Protection, Primary Care Trust Health Improvement Service and Community Dieticians (as was)  Dietician developed the criteria for award  City Council food safety officers issue awards |
| Award 5  On-going  Started in 2009 | To make **food that is good for people** and the planet the norm wherever people eat outside the home | *Award criteria:*  Intervention is compliance with a set of standards.  The standards categories are ‘making healthy eating easy’, ‘fresh food you can trust’, ‘championing local food producers’ and ‘sourcing environmentally sustainable and ethical food. | Chefs, nutritionists, public health experts involved in formulating the standards.  Independently audited by a national food charity on an annual basis to ensure compliance, and in doing so retain their award. |
| Award 6  On-going  Ongoing since 2005 | Reduce food borne infection  Reduce levels of obesity/ excess weight Increase food sustainability  Increase economic impact of Cornish food industry Recognise and reward caterers who adopt and maintain a high degree of food safety and standards as well as offering their customers **healthier options/choices** | *Award criteria:*  Demonstrate the highest possible standards of food hygiene, offer healthy options on their menus and source some Cornish ingredients in order to qualify for it.  Exact criteria for each level not reported | Environmental Health, Trading Standards, and a NHS food project  Award criteria written by qualified nutritionists and dieticians  Team also involves 2 representatives from the Industry  Awarded by the food safety and trading standards officers at the local Council |
| Award 7  On-going  Started March 2013 | To understand current foods and methods of cooking in local takeaway establishments.  To give businesses the opportunity by meeting the essential assessment criteria, to offer their customers food which has been prepared/cooked in a **healthier** manner and be awarded for this by means of a certificate and sticker. | *Award criteria:*  Meeting catering standards/ award scheme – focusing on: **Energy content; fat; salt and; sugar**.  Exact criteria not reported. | NHS Public Health Directorate, Trading Standards, local County Council, Environmental Health, local City Council, and Paediatrics and Public Health Community Services  Health improvement specialist, Specialist Dietitian, Environmental Health officers, Community and Business Support Officer |
| Award 8  On-going  July 2013 to July 2015 | Encourage takeaways to offer **healthier choices** and market their products to encourage their customers to **make healthier choices** | *Award criteria:*  The Awards are based around a number of healthy eating principles: Making food generally healthier by reducing **fat, salt and sugar** and increasing the use of **fruit and vegetables**. Designing catering practices, including food preparation and cooking methods, to produce healthier food. Helping consumers make informed choices, ensuring that wherever possible, information on food ingredients and cooking practices is made available. Portion sizes are appropriate, with options for smaller portions available. The active promotion of healthy eating, including pricing sales and marketing practices that encourage consumers to eat healthier foods  *Additional intervention:*  Free assessment on how to improve the standards of their catering practices, specific to their business. Takeaways are shown how they can make small changes to their recipes and menus to make their food healthier. Free training about nutrition awareness master-classes. Food samples taken to test for parameters such as **salt content, sugar, fatty acid profile and calorific value**. | Trading Standards and local Council including the public health and environmental health departments lead.  Principal dietitians delivered training to takeaway staff in a nutrition awareness master class.  Accredited by the local Council in partnership with Trading Standards and members of a Steering Group including Councillors, Public Health, Trading Standards, NHS and Environmental Health representation |
| Award 9  On-going  Started it in 2009 when won funding  The project as a brand was launched in 2011 | Recognise and reward food businesses that offer **healthy food** options and promote healthy eating  Part of a broad health improvement programme that aims to reduce local obesity rates and create a healthy town  Make food generally healthier and make it easier for customers to make informed choices when eating out | *Award criteria:*  Awards are based on the following six healthy eating principles: Reducing the amount of **fat, salt and sugar** and increasing the use of **fruit and vegetables**; Catering practices, including food preparation and cooking methods, designed to produce healthier food; Consumers are able to make an informed choice; Healthy and nutritious food is provided for children; Portion sizes are appropriate for a healthy diet; Healthy eating is actively promoted (includes pricing, sales and marketing practices)  *Additional intervention:*  Support has been provided to businesses wishing to improve the nutritional quality of their food with use of Saffron nutritional analysis software. | Local Council Environmental Health  The programme was developed with support from Nutritionists, Environmental Health and Public Health Officers.  Accredited by Environmental Health/Public Health Officers |
| Award 10  On-going  In operation since 1996; re-branded, revised and re-launched in 2005 | Improve access to as many healthy choices as possible for the consumer  Designed to complement the principles of the national healthy eating agenda of reducing **fat, salt and sugar** content as well as increasing **fibre, fruit and vegetable** consumption in line with the 5-A- Day initiative and change4life | *Award criteria:*  Level is dependent on the amount of points scored.  Exact criteria not reported.  Good practice examples include: food labelling and items displayed with calories; offering at least one reduced sugar item on their menu; offering lower sodium salt as an option at point of sale to customers; and ensuring the 5 A Day criteria is incorporated into menu’s. | Coordinated by Environmental Health within local Council Investment and Regeneration Directorate in partnership with the local Public Health Team (and sits within the Food, Safety & Wellbeing Team)  Environmental Health Officers and project advisors (who are qualified nutritionists)  Accredited by local Council and Public Health Team |
| Award 11  On-going  Mar 2013 – Mar 2015 | To support caterers to achieve the standards required for the award | *Award criteria:*  There are set criteria and a scoring tool used to assess outlets (criteria not reported)  *Additional intervention:*  Provision of training, supporting literature and resources, advice and recommendations on improvements to catering practices. | Jointly managed by the Food and Nutrition Team and the Environmental Health Team. |
| Award 12  On-going  Not reported | Encourage food businesses to reduce the amount of **salt, sugar and saturated** fat in their dishes and increase availability and consumption of **fruit and vegetables**.  To promote healthier cooking methods, correct proportions and serving sizes, and assists businesses to promote their healthy food options. | *Award criteria:*  Criteria not reported  *Additional intervention:*  Businesses provided with fact sheets with advice on how to improve their food  EHOs advise on any queries regarding cooking techniques, ingredient choices and recipes during assessment visit | Environmental health  Assessment and accreditation by EHO |
| Award 13  On-going  September 2013 to March 2015 (with possible extension, subject to evaluation of the pilot)  Launch date mid-end May 2014 | To transform the food environment of cafes, takeaways and restaurants  Have a beneficial effect on health and well-being by reducing the prevalence of ill health strongly associated with diet, whilst being mindful of the businesses to attract a strong customer base and turn a profit | *Award criteria:*  33% of their menus will provide **healthier options** | A general practitioner led organisation that delivers affordable and successful behavioural change programmes to improve public health) and  Public Health Team at a local Council  Team includes a Nutritionist |
| Award 14  On-going  Unclear | • Encourage caterers to think about healthier options e.g. less **salt, sugar and saturated fat**  • Reward caterers who make it easier for customers to eat healthily increased publicity  • Assist caterers in meeting the demand for healthier meals - advice, guidance and low cost nutrition training  • Help customers make informed choice e.g. by including healthier items on the menu which are clearly identified | *Award criteria:*  Scoring criteria based on healthier options available to customers and reducing the amount of **sugar, fat and salt** that is added to foods during cooking, preparation and serving, where possible. Criteria also encourages key members of staff to obtain a qualification in nutrition (ie; RSPH /CIEH Level 2 Award in healthier foods & special diets) to demonstrate their basic understanding of nutrition which is then shared with other staff members  *Additional intervention:*  Nutrition training part of award criteria | Local division of the Department of Health, local County Council Trading Standards, Environmental Health, Public Health, Health and Social Care Joint Training and a local University |
| Award 15  On-going  Pilot began in Spring 2008 | Not reported | *Award criteria:*  Criteria for each level not reported.  At each level, businesses have to show they are compliant with food safety, food standards, licensing and age restricted sales legislation and have an awareness of healthy eating, environmental issues, allergens and alcohol issues.  4 key areas: Clean and Fair; Allergen/ Alcohol Awareness; Healthy Eating; Children's menus  *Additional intervention:*  Advice on **healthier alternatives**, advice on how to improve the Food Hygiene Rating Scores, and Allergens given during assessment visit  Participating Businesses will receive a menu analysis | Local County Council Trading Standards administer the award  Environmental Health working in partnership with Public Health.  Nutrition experts in local Health Trusts  Assessment and accreditation by Public Protection Service (PPS) officers  Menu analysis carried out by dieticians |
| Award 16  On-going  Running for over 6 years, no end date | Recognise those catering businesses that have been assessed as implementing **healthier food** preparation methods and offering healthy eating options to consumers across the board | *Award criteria:*  Criteria not reported  *Additional intervention:*  A leaflet providing information and tips and advice from EHO | The scheme is run jointly by local Council and Primary Care Trust  EHOs with Level 2 nutrition training, plus NHS advice as appropriate |
| Award 17  On-going  Launched in summer 2008. End date for OHFO of interest 2014. | To improve the **healthy food** offerings of food establishments | *Award criteria:*  Food businesses have to have a hygiene rating equivalent to 3 stars or above  Assessment focuses on the following areas:  • Cooking and preparation  • Menu choice  • Promotion and marketing  • ‘Healthy Choice’ criteria  • Includes ‘Breastfeeding Welcome’  • Bronze - 35% ‘Healthy Choice’ options  • Silver - 50% ‘Healthy Choice’ options  • Gold - 75% ‘Healthy Choice’ options  *Additional intervention:*  Guidance packs available  Nutritionist analyses menus and clearly marks the Healthy Choice options. Where necessary, the nutritionist recommends menu changes.  Window sticker and free publicity via project website | Local Council with local Food Partnership and NHS Trust. Includes assessment by a nutritionist. |
| Award 18  On-going  Feb 2014 – end of March 2015 | • Adopt a **healthy catering** programme and sign-up food businesses to the voluntary code.  • Encourage caterers to make **healthy eating** easy and accessible for their customers by providing and promoting healthy food within their establishments  • Provide recognition to our catering businesses who have integrated **healthy eating** as a key consideration within menu provision and day-to-day practice. Businesses that show this commitment will be offered a window sticker to that effect.  • Increase consumer demand for **healthier options** when eating out of the home. | *Award criteria:*  Criteria not reported but follows simpler methodology than the one described for the Healthier Catering Commitment  *Additional intervention:*  Customer focused literature that describe the programme:  cover letter, questionnaire, marketing materials, stickers, certificates and leaflets  Support catering outlets to make the changes required for them to meet the criteria | Not reported |
| Award 19  On-going  Started May 2012  Pilot completed end of March 2014/early April | Contribute to creating an environment that supports and facilitates **healthier choices** by individuals and families | *Award criteria:*  Criteria not reported  *Additional intervention:*  Top Ten Tips leaflet for Businesses  Environmental Health staff who carry out food safety interventions will work with owners of food establishments and advise on healthier substitutes | District and borough councils, Environmental Health Officers and Trading Standards, local Food Liaison Group, local Obesity commissioning group, Community Dietetics and nutrition service (through working group) |
| Award 20  On-going  Start 2009 – no end date | To reward food businesses which offer **healthy options** on their menu and meet the minimum acceptable food hygiene standards | *Award criteria:*  Standard:  Must meet 11 criteria. Includes small realistic changes could make big differences, such as 1) reducing chip portions, 2) carrying out a “shake bang and hang technique” for fried food and 3) swapping a partially hydrogenated cooking oil for a liquid, low **saturated fat** type such as rapeseed.  Silver:  Meet all 11 standard criteria plus 10 further criteria. Included 1) providing at least one portion of **fruit and vegetables** 2) **wholemeal or wholegrain carbohydrates** 3) **oily fish** being available once a week and 4) providing a **carbohydrate** alternative to chips.  Gold:  Meet all 11 criteria for the standard, all of the 10 silver criteria and a then a further 4 criteria: 1) the Manager or Head Chef of food outlet must have undertaken nutrition training and/or healthy catering training 2) They must have adapted their recipes to make them healthier and 3) if they had vending machines, these must include healthier choices. The fourth and final criterion in this section was to be welcoming of mothers who wished to breastfeed  *Additional intervention:*  Training workshops available: healthy catering, healthier frying and practical cooking | The Local Authority’s Environmental Health Food Safety team were commissioned by Public Health to deliver the project  Team Lead EHO and Lead Public Health Dietitian  Volunteer Nutritionist currently studying MSc Dietetics |
| Award 21  On-going  October 2013 – date | To promote **healthier eating practices** by working with Food Business Operators to encourage change of menus and employ **healthier catering practices**. | Caterers can take many steps to provide healthier food choices for their customers by making healthier choices available, e.g. offering a choice of boiled or mashed potatoes as **alternatives to chips**; extra **vegetables** in servings; **fruit** based desserts and **wholemeal** bread choices with sandwiches. Reduction of portion size for children/elderly. Providing low **sugar** alternative drinks and tap water. Caterers can also prepare the food in healthier ways by reducing **salt**, trimming **fat** from meat before cooking, substituting unsaturated **fats** for **saturated fats**, and allowing customers to add as much or as little as they wish of sauces, dressings and fat spreads.  *Additional intervention:*  Local specialist catering suppliers were approached to supply reduced salt/sugar on demand from FBO’s.  Window stickers given out for successful food businesses. Merit logos shown on website alongside Food Hygiene rating and report on county council website. | Local City, District and Borough Councils, working with local County Council Public Health.  Inter disciplinary work between Environmental Health Practitioners and publicity funding from the Public Health Team at the County Council.  Advice from nutritionist when setting original criteria |
| Award 22  On-going  Not reported | Recognise and help those businesses who want to provide safe, healthy environments and **healthy food** choices for customers and staff.  Help consumers to identify food outlets where healthy choices are made available. | *Award criteria:*  Businesses must demonstrate that they provide healthy choices for their customers by reducing **fats, salts and sugars** in your food preparation and service, wherever possible, whilst using opportunities to promote **fruit, salads and vegetables**.  *Additional intervention:*  Initially information and advice on how to apply and achieve the standards required provided.  During assessment, latest public health resources and information and tips on adjustments and improvements that they can make to their menu provided.  information and signposting event consisting of talks on portion control, a cookery demonstration and various organisations in attendance to give advice and information including the British Heart Foundation, local leisure centres, private healthcare providers, fruit and vegetable distributors and local supermarkets. | Health Team and Food and Commercial Team at local Borough Council  Environmental Health Officers inspecting businesses actively promote the awards  A nutritionist carries out the assessment |
| Award 23  On-going  Started in 1990 | To promote appropriate healthy eating in standard food outlets, homes caring for vulnerable adults and nurseries; To offer of a range of **healthy food choices** to diners; To promote healthy choices to diners | *Award criteria:*  Businesses must demonstrate that they provide healthy food options under the following headings: **Fruit and Vegetables; Bread, other cereals and potato; Milk & dairy produce; Meat, fish and alternatives; Fat, sugar and salt**; Portion sizes; Display pricing and marketing. To reach the standard for the healthy award, it should be easy for customers to select healthy foods from a menu / serving area. If there is no choice, the only option should be a healthy one. | Local Environmental Health Department lead.  Award run by local Councils and NHS dietitians.  Promoted by EHOs. Menus assessed by dietitian. |
| Award 24  On-going  Launched February 2014 | Reduce **saturated fat** content of chips served in fish and chip takeaways | *Award criteria:*  Focuses on chip shops frying at higher temperatures and following processes that helps to maintain their oil from premature degradation  *Additional intervention:*  If awarded, businesses are issued with a window sticker (along with certificate and a supply of oil quality test strips to help them monitor oil degradation  If unsuccessful, guidance and training will take place so that business can work towards accreditation | Local Council, Environmental Health Section and Public Health team |
| Award 25  Completed  April 2010 – October 2011 | To encourage small independent businesses preparing sandwiches & snacks to make some simple changes to their ingredients and preparation methods in order to produce an overall **healthier range of products** that are lower in **saturated fat, sugar and salt** and without impacting on bottom line. | *Award criteria:*  Award was given to those businesses who stated they would be taking all the agreed minimum changes  Examples of changes:  o Offering different types of bread - including wholemeal/granary  o Offering a low fat spread or using no spread at all  o Offering salad with every sandwich  o Offering healthier snacks & drinks  o Using lower fat mayonnaise  o Promoting the healthier options  *Additional intervention:*  Guidance ‘Tips’ for Healthier Sandwiches  Business promoted on website | The Food Safety Team of the local City Council led this work.  Partners:  Food Safety Team, Dietician working with City Council’s School Meals project, Health Policy Lead – local City Council  Supported by ‘Food & Health Strategy’ group (joint working group NHS/City Council) and Change4Life |
| Award 26 ([Holdsworth et al., 1997](#_ENREF_3), [Warm et al., 1997](#_ENREF_4))  Completed  Started April 1990 – end date unclear | To reduce total **fat, sugar, and salt** and increase the availability of **fibre-rich, starchy foods.** | *Award criteria:*  - At least one-third of the meals on the menu should be healthy choices (defined as low in fat and rich in starch and fibre)  - At least one-third of the seating should be designated as non-smoking  - At least one-third of the food-handling staff should have received food hygiene training  *Additional intervention:*  The award scheme is marketed in establishments using posters, leaflets, and symbols next to healthier food options.  Advice on recipe modification and the promotion of healthier food choices provided | Health Education Authority and Institution of Environmental Health Officers; in 1991 HEA passed over control to local authorities for the running of the award  EHOs responsible for accrediting the award  Community dietitian assessed the menus and offered advice on recipe modification and the promotion of healthier food choices |
| Award 27 ([Hanratty et al., 2012](#_ENREF_2))  Completed  Not reported | Unclear | *Award criteria:*  Businesses that demonstrate that they were offering at least one **healthy option** on their menus, along with a range of healthy eating criteria  Exact criteria unclear | PCT and LA public health workers |
| Award 28  Completed  Pre 2009 to 2012 | To recognise caterers who are committed to offering **healthier eating choices** | *Award criteria:*  Comprehensive checklist determines level of award but exact criteria not reported.  Award covers 4 criteria: Compliance with food hygiene regulations, including training; Promotion and provision of healthier food choices (questions relating to **fruit & vegetables, protein, fibre, dairy, oils & spreads, salt,** cooking methods & food preparation, serving food, drinks, snacks, children’s meals and supporting healthier eating); Compliance with smoke-free requirements; and Breastfeeding friendly policy.  *Additional intervention:*  In addition to certificate, businesses receive a window logo, regular newsletter, media promotion, free information and support on request, and ongoing recognition of commitment to healthier eating | Partnership between local Environmental Health Departments and two local PCTs  Award assessor accredits award – expertise unclear |
| Award 29  Completed  December 2007 – April 2011 Launched officially in April 2009 | To have a positive impact on the health of the borough’s residents by increasing access to food lower in **fat, saturated fat, salt and sugar** | *Award criteria:*  The nutritional improvement required by each business type varied according to the audience that the business catered for and their needs. The common approach was to make the food served more nutritionally balanced and tackle the  following areas:  • Reduce added salt and that added through high salt ingredients e.g. stocks and gravies and reduce processed food.  • Eliminate trans-fat through hydrogenated vegetable oils.  • Reduce the energy density of foods (except elderly requiring more energy dense diets).  • Reduce fat especially saturated fat through cooking practices, recipe reformulation and offering products without added fat enrichment e.g. butter on sandwiches.  • Increase fruit and veg either through bulking dishes or making fruit and veg more available.  • Increase fibre intake through wholegrains.  *Additional intervention:*  The healthier dishes were labelled with a logo | The programme is delivered by a multidisciplinary team that include specialists in nutrition, food safety, trading standards, food science and engineering, catering and communications |
| Award 30  Completed  Start date not specified. Pilot completed December 2008. The main project finished in 2011. | To improve the health of residents by increasing the **healthy eating choices** available to them.  To empower people to make informed **healthy eating choices** | *Award criteria:*  Criteria not reported  *Additional intervention:*  Each café was given advice from a nutritionist about healthy eating and how they could make small changes to their menus to ensure their customers were given **healthier options**, but without an increase in cost.  Support given for café to access suitable training /chef trainer visited cafes | Partnership between a local food access partnership, a health charity and an existing community health project.  Nutritionist and chef trainer |
| Award 31  Completed  2008 – 2012 | • To make the **food served generally healthier** by making broad changes to how food is prepared. • To help customers make informed choices about food by including healthier items on the menu which are clearly identified. | *Award criteria:*  Nutritional requirements:  Keep the level of **fats and oils** to a minimum in the food you serve, in particular **saturated fat**; Keep the level of **salt** to a minimum in the food you serve; Keep the level of sugar to a minimum in the food you serve; Make **fruit and vegetables** clearly available; Make **starchy foods** the main part of most meals; Make sure that at least 50% of the foods served are labelled as healthy choices, which are prepared using both healthier ingredients and cooking methods; Have an appropriate sales promotion and marketing strategy which works alongside the general principles of the award and supports healthier eating  In addition to food hygiene, smoking and breastfeeding requirements  *Additional intervention:*  Useful nutrition information in project booklet.  Free resources are available to help Food Providers promote their establishment as an award holder and to promote the healthy options on your menu.  In addition the Food Providers are provided with branded resources to advertise their success. | Award was managed by the health development team within the local Borough Council. Assessments conducted by a team of professional  healthy eating specialists  Staff were guided by community nutritionist and healthy eating co-ordinator as to best practice |
| Award 32  Unclear  Unclear | To recognise those catering establishments who are working towards making the **food they offer healthier** and are promoting those **healthier options** | *Award criteria:*  Commitment Award  This award certifies that this catering establishment:  • Has achieved a food hygiene rating score of at least 3  • Is committed to offering a range of food and drinks to their customers that are lower in **saturated fat, trans fat, sugar and/or salt**  • Will offer a portion of **fruit or vegetables**.  Achievement Award  Same as Commitment Award except that catering establishment:  • Has achieved a food hygiene rating score of at least 4.  • Will actively promote healthier foods and drinks to their customers.  Excellence Award  Same as Achievement Award except that catering establishment:  • Has achieved a food hygiene rating score of 5.  • Will have in place strategies to promote sustainability.  *Additional intervention:*  In addition to certificate, the business will receive a window sticker, award logo for their website and a place on the Healthier Catering website. | The scheme was devised by the local Programme Manager for Healthy Weight, a registered dietitian, a nutrition coordinator at a local council (registered nutritionist), and the Food Safety Manager a second local Council (Environmental Health Officer) |
| Award 33  Unclear  Unclear | To make it easier for local people to find a food outlet that offers and promotes **healthier options** | *Award criteria:*  Criteria not reported  *Additional intervention:*  Staff from the community food and health team work with the staff of the businesses to help them promote healthier eating choices for customers. | Run by the local community food and health team, part of the local NHS Foundation Trust. The Trust works in partnership with the Council’s Environmental Health team.  Award assessed and accredited by  environmental health and the food and health team |
| Award 34 (HCC) ([Bagwell, 2014](#_ENREF_1))  On-going  Pilot was launched formally on 14 March 2012  Data for evaluation collected in March/April 2012 | Encourage businesses in the catering trade to reduce the level of **saturated fat and sugar** content of foods, to offer healthier options and/or smaller portions and adopt **healthier cooking practices**. It also aims to raise awareness of the importance of providing healthier food choices and ideally lead to changes in consumption behaviour. | *Award criteria:* See overall Healthy Catering Commitment award initiative (HCC) criteria^2^ | Chartered Institute for Environmental Health (CIEH), the Association of London Environmental Health Managers (ALEHM), and the Greater London Authority (GLA)  Designed to be implemented and accredited by Environmental Health Officers.  Boroughs implementing the scheme are free to adopt a delivery approach that best suits their local priorities |
| Award 35 (HCC)  On-going  Not stated | 1. Encourage and provide food businesses with guidance that will enable them to reduce the level of **saturated fat, salt and sugar** content of foods, to offer healthier options and/or smaller portions and adopt healthier cooking practices.  2. To raise awareness of the importance of providing healthier food choices and that could lead to changes in consumption behaviour. | *Award criteria:* See overall HCC criteria^2^  *Additional intervention:*  Some initial analysis of the food samples from the outlets has been done and revealed the levels of salt, fat and sugar in some of the main dishes that featured on their menu. Based on the findings and working with the businesses suggestions have been made to make these and other dishes on the menu more healthy. | Local Food Health Safety Team with assistance from the local Public Health Team  Public Analyst supported with sampling. Commissioned piece of work with nutritionist following sample results. Students from Public Health worked to develop the leaflets for community businesses. |
| Award 36 (HCC)  On-going  Nov 2013 – March 2015 | To promote and increase the take up of HCC in Harrow | *Award criteria:* See overall HCC criteria^2^  *Additional intervention:*  A Master Class workshop will cover a basic nutrition with healthier cooking, the HCC and participation of the FoodSave company. Offered to food establishments that already obtained a good food hygiene rating but have not signed up for the HCC. | Environmental Health and Public Health teams in the local Council |
| Award 37 (HCC)  On-going  2012 - date | To recognise food outlets which offer **healthier food options** | *Award criteria:* See overall HCC criteria^1^ | Local council (Implemented by Food Safety team) in partnership with the Greater London Authority, local NHS and the Chartered Institute of Environmental Health. |
| Award 38 (HCC)  On-going  2009/10 – date |  | *Award criteria:* See overall HCC criteria^2^ | Public Health and the Environmental Health Team  Accredited by the Food Safety team at the local Council |
| Award 39 (HCC)  On-going  2013 - date | The HCC scheme aims to encourage businesses to reduce the levels of **saturated fat, salt and sugar** in foods, offer healthier options and/or smaller portions and adopt healthier cooking practices | *Award criteria:* See overall HCC criteria^2^  *Additional intervention:*  Planned: Workshops on ‘frying’ and nutrition, and provision of free ‘five hole salt shakers’ | Local Council Environmental Health and Public Health  Environmental Health/Food Safety Officers carry out assessments |
| Award 40 (HCC)  On-going  2012-2014 | To encourage businesses in the catering trade to reduce the level of **saturated fat, salt and sugar** content of foods, to offer healthier options and/or smaller portions and adopt healthier cooking and preparation practices by using the ‘small changes make a big difference’ principle.  To raise awareness of the importance of providing healthier food choices and ideally lead to changes in consumption behaviour. | *Award criteria:* See overall HCC criteria^2^  *Additional intervention:*  K&C - support the development of a Healthy Menu | Local Environmental Health team, Public Health Nutrition teams and Primary Care Trust (funders), and a local university (evaluation) |
| Award 41 (HCC)  Completed  January 2012 to November 2013 | • Work with local businesses to create health pledges that adhered to the SMART guidelines  • Promote **healthy eating**, alcohol awareness, workplace health and physical activity  • Provide businesses with examples of pledges in order to increase health initiatives  • Offer rewards (i.e., recognition, certificates) for participating in pledges  • Increase interest in health promotion | *Award criteria:* See overall HCC criteria^2^ | Public Health Nutritionist and  Environmental Health Officers |
| Award 42 (HCC)  Completed  February – May 2012 | To support businesses to achieve the Healthier Catering Commitment (HCC) for their food and drink provision across their whole menu  To develop promotional/publicity materials for the businesses for the advertisement of HCC  To evidence an approach for a wider rollout. | *Award criteria:* See overall HCC criteria^2^  *Additional intervention:*  Promotional materials supporting branding of HCC and complement EC regulation for nutrition and health claims made on foods. Included table top A5 menu, A3 street facing poster plus Change4Life table-talkers (on swaps) | Registered Nutritionist & local Environmental Health |
| Award 43 (HCC)  Completed  October 2012 – February 2013 | To support businesses to achieve the Healthier Catering Commitment (HCC) for their food and drink provision across their whole menu | *Award criteria:* See overall HCC criteria^2^  *Additional intervention:*  Promotional materials supporting branding of HCC and complement EC regulation for nutrition and health claims made on foods. Included table top A5 menu, A3 street facing poster plus Change4Life table-talkers (on swaps | Registered Nutritionist & local Environmental Health |
| Non-award 1  Planned  March 2014 – May 2015 | Set up an extensive network of street food traders serving tasty **food that meets health guidelines** at locally competitive prices, through a number of designated pitches located near secondary schools and large numbers of unhealthy fast food outlets. | To secure a pitch, traders must be able to prove that their food meets the Department of Health’s nutritional guidelines, is popular with young people and costs under £3 for a meal. We will assist them in the nutritional testing and menu refinement process, and provide opportunities for them to test their food at events with local young people.  An overarching name and brand for the network and create a public facing identity will be developed.  Between 3 and 6 eight week training schemes per location, will be offered to an unemployed young person from the borough. These catering assistants will obtain a City and Guilds in Level 2 Food Safety and Hygiene. | A not-for-profit company involved in tackling social and environmental issues, backed with support from an organisation that produces and commissions arts projects in the area.  Consultations made with the key mobile food networks |
| Non-award 2  Planned  Not reported | Give caterers basic information on how to reduce their **salt, fat** etc when they are preparing food in their businesses | Currently putting together some healthy catering inserts to go into the national food safety pack called Safer Food Better Business which is already used by the majority of food businesses – very early stages of development | Not reported |
| Non-award 3  Planned  2014 – unknown | To increase the amount of **healthy food** eaten by secondary school children during the school day | Project team will work with local takeaway food businesses to reduce the amount of salt and fat in their products  Project team will encourage takeaway food businesses within 400m of the school to offer healthy options/meal deals to encourage pupils to make a healthier choice | Local City Council Public Protection and Public Health departments  City Council Principal Health Improvement Officer |
| Non-award 4  Planned  Unclear | Work with fast food outlets to improve the nutritional composition of food based on the London Healthier Catering model | Focus on working with takeaways around their frying practice, which has the biggest impact in terms of **reducing fat and calories** intake, was relatively easy for takeaways to adopt, and could potentially help them save money. Takeaways could also make other changes around **fats and oils, salt, sugar and fruit and vegetables**. | Public Health Nutrition Team, Nutrition and Dietetic Service, and local City Care Partnership.  Specialist Public Health Nutritionist  Community Nutritionist  Community Food Workers. |
| Non-award 5  Planned  April 2014 – March 2015 (hope to extend the project for another 2 years depending on agreement and/or funding) | Work with takeaways to improve the healthy food offered; Tackle over weight and obesity in the local population; Think positively about food as a means of feeling good and good health | Pilot the Mayor of London’s /Chartered Institute of Environmental Health takeaways toolkit with a number of food businesses.  Intervention/ support offered will depend on outcomes of initial consultations with the outlets.  May produce educational materials and/or develop a local award scheme.  Also aim to create a demand from the customer side by creating an awareness of **salt/sugar/fat** content. | Local City Council  Environmental Health & Trading Standards together with Public Health.  Also hope to gain some assistance from communications team and tender out some work to a local VCF provider with a proven track record in this area. |
| Non-award 6  On-going  July 2013 – May 2014 | To undertake interventions in ten targeted Chip Shop/Kebab takeaway premises | Interventions were broken down into the following areas: **Salt, Fat, Sugar**, Labelling, Portion Size, Healthier Cooking Techniques  Visit from a Public Protection Officer who assesses the businesses on a number of criteria relating to current catering practices that affect **salt, fat and sugar** content, availability (and promotion) of healthier options. Some of the assessment is based on the Food Standards Agency ‘Tips on Chips’.  Businesses receive an information pack and a letter with recommendations and then premises is re-visited and re-assessed, following the same criteria, 6 to 8 weeks later. | Local Council (Health Protection & Prevention Team – with Environmental Health background) |
| Non-award 7  On-going  2013 – date | To improve the standard of food and service offered to children when they eat out in restaurants, pubs and cafes, focusing on providing food variety and making **healthy eating** easy, making sure children’s needs are accommodate for and that food is cooked fresh from good quality ingredients | The key calls to action are to:  • Offer all children the choice of a child's portion of adult meals  • Serve freshly prepared food, not ready meals  • Offer free **water** to all families on arrival  • Offer children’s cutlery as standard  • Make breastfeeding mums feel welcome  • Make sure all main meals contain a portion of **vegetables** and some puddings include a portion of **fruit**  A league table displayed on the food charity’s website based on information from three sources:    1. Online menus on restaurant and pub websites  2. A survey of all establishments  3. A field survey of establishments conducted by parents across the UK  Work with leading chains to improve their practices, with a view to benchmarking their achievements with another league table in 2015.  A toolkit is being promoted to all restaurants, pubs and cafes to support them to improve their practices. | National food charity  The criteria and key calls to action were consulted on with public health experts, nutritionists and catering stakeholders |
| Non-award 8  On-going  October 2013 – October 2014 | •To identify business models that enable independent fast food outlets to adopt **healthier menus and catering practices** without compromising the profitability of their business. •To enable public health officials to have a better understanding of the operational barriers which may make it difficult for businesses to make healthier changes. •To work with businesses and public health officials to identify further behaviour change strategies that can successfully be used by fast food businesses to encourage healthier consumption patterns amongst their customers. •To work with suppliers of independent fast food outlets to identify ways in which products can be reformulated or promoted to encourage businesses to purchase healthier varieties. | •A telephone survey of healthy catering initiatives and work with suppliers conducted elsewhere to identify good practice and lessons learnt. •Detailed interviews with fast food outlets to provide examples of good practice and/or trial changes, nudges etc. to enable an in-depth understanding of the business to be developed including its products/menus, market and marketing strategy, as well as barriers to the introduction of healthier changes. •Work with suppliers to encourage product reformulation and new marketing strategies designed to encourage the promotion and sale of healthier varieties of products. Outputs from the project will include a number of resources for public sector practitioners including: •The development of a number of case studies of businesses that have attempted to adopt new business models to include menu and product reformulation to offer financially viable healthier catering. •Suggested healthier behaviour change strategies or ‘nudges’ that businesses can be encouraged to adopt. •A guide for suppliers to include recommendations for product, packaging, and marketing strategies that are likely to lead to the sale and purchase of healthier products and portions. •A briefing paper on key recommendations for policy. •These all to form part of a web-based best practice toolkit located on the CIEH website. •Dissemination events, held in conjunction with the GLA, and the CIEH will promote the toolkit to practitioners and policy makers throughout the UK. | A local university, Healthy Catering Commitment Network, Association of Environmental Health Managers and GLA Food Team |
| Non-award 9  On-going  2007 – date | • To modify and produce very popular meals in the **healthiest** possible way  • To create a market for project branded meals so that the project can continue well after the funding has ceased.  • To create a demand for **nutritionally improved meals** from both consumers and the trade which will, hopefully, lead to the production and availability of nutritionally improved catering ingredients, produced by food manufacturers • To contribute towards the local PCT’s aim to cut deaths from Cardio Vascular Disease by half in under - 75 year olds, and to contribute to other national indicators relating to obesity, cancers, and life expectancy • For the city to be seen as synonymous with healthy eating in the food away from home sector and for the project to be used as a model for best practice. | Recipes were collected from pilot outlets-involved watching the chef cook each dish and weighing out and recording each ingredient. Samples of the meals were nutritionally analysed. LJMU reformulated the recipes. New recipes were cooked in the outlets and tasted. Small adjustments to the recipe happened in the outlet. Once the outlet/chef was happy with the new recipe this was cooked and sent for nutritional analysis. Once on sale in the outlet further samples taken to ensure reductions in fat and salt still being obtained.  5 hole salt shakers offered to every takeaway  Resource Pack written and developed  Free training for catering staff – basic nutrition and food hygiene (this part if project ended in 2011)  Certificate of participation given to businesses and recognition on website | Public Health department, Trading Standards department, and a local University.  Partners: The City Chinese Business Association and the Muslim Enterprise Development Service  TSD is responsible for the overall management of the project.  Local university - research into recipe development and evaluation (nutritionist/chef - working with the TSD and participating establishments |
| Non-award 10  On-going  2011 – 2014 | Build a robust evidence base of nutritional information which will; Allow comparison on a local, regional and national level; Allow comparison to the Local Government Regulation survey; Allow comparison with local health data around mortality and hospital admission rates for poor nutritional related illness; Contribute to a pool of city-wide intelligence being developed around takeaway outlets; Provide baseline data to allow future analysis work to be undertaken and any changes monitored. Develop an action plan which will include advisory, health promotion and enforcement activities, along with suggested changes to local policy. Support appropriate action around takeaway food outlets on a local, city-wide and national level. obtained levels of trans-fats, sugars and calories within samples | Nutritional sampling of food from every hot food takeaway outlet within the borough  Results feedback to businesses  Training will be provided to owners, chefs and staff of outlets (final phase).  Sampling will be repeated after training. | Local Council (and local NHS, now disbanded). Support also provided by a local CVD Charity.  Environmental Health Officers and nutritionist |
| Non-award 11  On-going  Unclear but on-going January 2013 | To encourage takeaways (Asian food) to provide **calorific values** of foods on their menus and create some **healthier options** | Calorie labelling and reformulation  No further details provided | Local City Council (Principal Health Improvement Officer) |
| Non-award 12  On-going  Unclear | Not reported | Local Authority have worked with the Café to adapt popular choices to **healthier versions**, deliver staff training (CEIH level 2 - nutrition and food hygiene) and create menu sign posting. | Local Authority (Environmental Health) |
| Non-award 13  On-going  Not clear | Improve the skills of workers in Luton’s catering establishments; encourage catering establishments in Luton to provide **healthier food** by reducing **saturated fat** used in meal preparation | Training element: improving hygiene skills in kitchens initiative  The healthy eating element: Collect baseline data on the type of fats being used in catering premises (brands); Create a database of nutritional information from the baseline information; Develop a traffic light system to identify healthier brands of fat for caterers; Select target area and promote as their responsibility within the community – potentially an area with a high prevalence of obesity and health inequalities; Review the percentage of caterers changing brand use. | Health Improvement Specialist  Public Health |
| Non-award 14  On-going  January 2014 – March 2015 | To reduce **fat, salt, sugar and calories** of takeaway food as well as increase **fruit and vegetable** consumption. | Free ‘takeaway master class’ which will provide training around healthy eating messages and practices.  Training will cover food preparation habits, food ingredients used and cooking techniques. Guest expert speaker will also provide advice on healthier frying techniques including the use of different oils to maintain oil quality, taste and save businesses money.  Takeaways will be awarded with a certificate to display on their premises and will be encouraged to introduce good practice and promote this to customers through healthy choice options on their menus. | Local Council’s Food Safety Team, local food initiative project team (including 2 nutritionists) & Federation of Fish Fryer’s |
| Non-award 15  Completed  August 2012 –May 2013 | To influence food choices towards **healthier options** at caterers operating mobile services without impacting on the business's bottom line | Initial visit to register their interest in the project. A total of six visits were planned for each premises.  Visits included taking samples of the most popular meals (analysed for **salt, carbohydrates, protein and fat** content); explaining results of sampling; top tips recommended and discussed.  The catering establishments were only asked to deliver the intervention for a four week period.  A food and equipment pack and a guidance pack were given to assist the proprietor in implementing the top tips | Delivered by one local Regulatory Services. The project was run in partnership with another local Regulatory Services, and funded by a local NHS Public Health team  The Top Tips were developed in conjunction with a nutritionist |
| Non-award 16  Completed  2012 – 2013 | To influence food choices towards **healthier options** at caterers operating mobile services without impacting on the business's bottom line | 1. Provide training, promotional material and facilitating the Change 4 Life brand  2. Improve **nutritional content** of menus  3. Reduce portion size  4. Make fresh **fruit** and **healthier options** available  5. Relocate visible unhealthy options from the till point.  Based on Worcestershire Truckers Tucker  Unclear if nutritional analysis of food samples was conducted | Local Authority Trading Standards Departments  Nutritionist |
| Non-award 17  Completed  November 2012 – November 2013 | To influence food choices towards **healthier catering/options** and to increase the **nutritional quality of food** items, and improve ‘health by stealth’. | Visit from Environmental Health Team who carries out an initial assessment. Feedback and recommendations are provided, and then officer re-visits after 6-8 weeks to evaluate progress.  Where appropriate, salt shaker with fewer holes and oil pump sprayers provided.  A certificate poster was provided to the food business to help promote healthy options. | Local Council (Environmental Health Team) |
| Non-award 18  Completed  January – March 2012 | ■ Introduce healthier menu options and more fresh **fruit**  ■ Improve the **nutritional values** of meals generally  ■ Relocate visible unhealthy options from the till point, and  ■ Provide training and promotional material to support Change 4 Life. | Trading Standards officers visited business over a period of 4 to 5 weeks to support it to change the menu they offer to include healthier choices  Various recipes provided to business; Use of low fat alternatives encouraged (in first instance supplied in the Truckers Tucker pack); Businesses encouraged to provide fresh fruit to customers and provided with a fruit bowl to hold the fruit; Set portions/ smaller portions encouraged by provision of chip scoop; Various helpful leaflets provided | Trading standards officers |
| Non-award 19  Completed  August 2009 – June 2011 | 1. Bring in a chef and experts to work with restaurants to change cooking practices and reformulate food so it has reduced **saturated fat, salt and calories** in popular takeaway dishes.  2. Work with restaurants to design new healthier options that taste as good – or better - than the higher **fat** equivalents.  3. Introduce new menus (and symbols) so consumers are aware of new healthier options.  4. Encourage the purchasing and use of smaller cartons to decrease portion sizes.  5. Work with suppliers to Indian restaurants to **reduce salt, sugar, saturated fat and calories** in ‘bought products’.  6. Raise awareness about takeaway food and encourage consumers to ask for **healthier options** in their local restaurant/ takeaway (thus encouraging businesses to develop alternatives to meet the needs of their customers).  7. Encourage restaurants and takeaways to ‘swap’ products for **healthier alternatives** (such as vegetable oil for rapeseed oil) | Guidance was offered to each restaurant & guide produced | Local NHS, two local County Councils, one District Council, the Food Standards Agency, a local Food Liaison Group and the regional Public Health Observatory |
| Non award 20  Completed  July 2009 – December 2011 | To change the behaviour of those who supply takeaway food to consumers such as cooks, food preparers and takeaway owners in a bid to provide **healthier food** options for consumers. | The supply chain was explored – how food was prepared, what was bought and where it was sourced from. The project also explored the competition and the exchange that is needed to get establishments to cut the high levels of saturated fat, salt and calories found in popular dishes. | Regional Government Office – Public Health, the Food Standards Agency, local NHS, local County Council, Consumer Direct, Trading Standards, National Social Marketing Centre and a local food liaison group |
| Non-award 21  Completed  July 2009 – January 2011 | To improve the **nutritional quality** of children’s food when eating out in the county | Series of training seminars on nutrition and healthier catering (based on Change4Life principles and FSA guidance) – delivered by nutrition consultant and attended by Environmental Health Officers. One-to-one consultation with nutrition consultant and EHO responsible for the business available for approximately half of the participating businesses – aimed to provide more targeted advice and practical tips on options for healthier catering in the context of their particular businesses, taking into account its nature, goals and individual constraints. One business per district could request a free nutrient analysis of their menus, before and after changes were made.  Free local publicity | Environmental Health Officers – supported by local NHS and County Council. Independent nutrition consultant |
| Non-award 22  Completed  October 2013 – March 2014 | To encourage behavioural change within families and to make them aware of **healthy alternatives** available from takeaways. | Grants given to allow the purchase of healthier frying oils, upgraded menus and signs to include healthy options, a healthy drink range, fewer holed salt shakers and new chipper blades.  Families and children were invited to attend healthy eating workshops to inform them about healthy diets and guide them on healthier options and swops that can be made. | Support from Environmental Health, Public Health, NHS and a local university |
| Non-award 23  Completed  September – October 2013 | Assess whether it was possible to introduce a new option into a landscape dominated by fast food outlets and create an enterprise that was **healthy**, popular and financially sustainable. | Mobile food unit set up selling cheap, **nutritious food**, marketed at a young audience with no interest in healthy eating.  The project also offered an 8 week training scheme to an unemployed young person, plus an 8 week internship to a university student as part of their business degree. These two catering assistants and the WAWWD Account Director obtained a City and Guilds in Level 2 Food Safety and Hygiene. | A not-for-profit company involved in tackling social and environmental issues, backed with support from an organisation that produces and commissions arts projects in the area. |
| Non-award 24  Completed  Started in 2010  End date not reported | To nutritionally analyse a cross-section of dishes from menus from takeaway outlets, located near to Secondary Schools and make recommendations with an aim to reduce **energy, fat (saturated fat), salt and sugar** i.e. increasing the availability of healthier options. | Each outlet received three visits from the nutritionist (some accompanied by Community dietitian).First visit to collect data on menus, ingredients/labels for analysis of food items (Saffron nutrient analysis system-computer programme), second to discuss results and plan for implementation of tailored (to the outlet) recommendations on e.g.reducing sugar, increasing 5-a-day, reduce salt, reduce fat/saturated fat. Salt shakers(with fewer holes) had been purchased and were distributed at this visit. This second visit was followed by a letter, highlighting a summary of the discussion and recommendations. Third visit (after 6-8 weeks) to evaluate the recommendations. A verbal evaluation was taken, to ascertain what changes were made, their impact, and any customer feedback. | Local PCT (Public Health) and Environmental Health, with project team members represented from  Public Health, Community Services, Catering Services, and Environmental  Health.  Public Health Nutritionist, Registered dietitian, Environmental health officer, Public Health manager |
| Non-award 25  Completed  2010 – May 2013 | To reduce the amount of **salt and fat** in the end product or foods sold from traditional fish and chip shops  To contribute to key national indicators concerning health improvements and reductions in obesity | Provision of advice pack on reformulation of recipes (**reducing salt and fat**), improved cooking techniques to reduce **fat**. Marketing of **water** and **sugar** free drinks.  Provision of 5 holed salt shaker. | Local City Council Public Protection involving Environmental Health Officers and Trading Standards  Principal Health Improvement Officer |
| Non-award 26  Completed  Start date unclear – 2010 | To reduce added **salt** at fish and chip shops through the distribution of five holed salt shakers, therefore reducing **salt** intake  • To engage and involve local food producers in demonstrating how they can make a difference and improve the health of their customers  • To promote salt reduction to consumers | Officers visited all fish and chip shops across the County using a trader checklist explaining the project, distributing new salt shakers, trader flyers and posters.  The poster stated the shop was using the healthy salt shaker and “This shop is supporting [the local] County Council in its work to improve the health and well being of [the county’s] residents”.  Press release with support from local traders and also the PCTs launched the project. | Local County Council Trading Standards and Partners (NHS Community Nutrition team; the local hospital Department of Nutrition and Dietetics; a Salt shaker manufacturer; and Fish and chip shop owners) |
| Non-award 27  Completed  2005 – 2007 | Reduce **salt** intake of fish and chip takeaway customers | Businesses were provided with free new pot lids for salt shakers designed by the officers with 5 holes instead of the normal 17 (a reduction of over 60% salt). Advisory leaflets about the importance of reducing the amount of salt on food were also given to the businesses, along with a poster to display in their shops (designed by the Food Control Team) to inform customers about using less salt | Local Council  Environmental Health Officers |
| Non-award 28  Completed  June – August 2010 | To attempt to influence food choices towards **healthier options** at independent sandwich shops without impacting on bottom line | Lead officer and nutritionist made an initial pre-visit to capture baseline data using a ‘pre-questionnaire’. The nutritionist then introduced the FSA’s guidance document, ‘sandwich tips’ and attempted to negotiate healthier changes that the shop would trial over the following month. A list of agreed actions was subsequently sent to each shop. Most of the changes made related to reducing the amount of saturated fat and salt in the sandwiches. | Environmental Health and Licensing Manager (local City Council)  Nutritionist |
| Non-award 29  Completed  2002 | • To increase the number of retailers providing **healthier “take-away” alternatives**;  • To increase the demand for **healthier “take-away” alternatives**. | 1. The partnership contacted all the small sandwich manufacturers and retailers in the region; 2. Current practice established via questionnaires; 3. A reduced fat mayonnaise at similar price to full fat negotiated with one local supplier; 4. During routine inspections by TS and EH, sandwich manufacturers were asked for their input in methods to advertise the message that sandwiches could be healthier if consumers request for no butter and low fat mayonnaise; 5. Posters, advice leaflets for retailers and credit card sized guidance for consumers were distributed; 5. Scheme publicised to consumers at the County Show and several articles were placed in local papers and advertised on regional news;  7. Pilot project: two sandwich manufacturers agreed to pilot a project for 6 months, during which time they did not use any margarine/spread and used low fat mayonnaise, however these facts were not made known to the customers; 8. Training seminars delivered with presentations were given by TS, EH and PCT. | A Food Partnership was formed consisting of Environmental Health departments, Primary Care Trust and Trading Standards officers across the county.  County Public Health Nutritionist based within the PCT’s was a key member of the Food Partnership team. |
| Non-award 30  Completed  October 2010 – March 2011 | To provide residents and customers with access to **healthier, more balanced choices** of fast food options alongside the food and drink purveyors already served at their takeaways. | Businesses chose to implement changes from a list of recommendations grouped into three categories: portion size, nutritional quality and variety of menu. The recommendations made were specific to the type of food on offer in the different takeaways and were all highlighted by adopting the My Choice logo to the recommended options. | Single provider Registered Nutritionist. The project was part of a local Community Project, which was funded by the local NHS, and directed by the local Community Project Steering Group. |
| Non-award 31  Completed  2009 – unclear | To develop a voluntary **calorie** labelling scheme for the catering industry that is clear, effective and simple to understand. To enable the process evaluation to take place twenty one participating | Guidance provided to participating companies in the form of criteria for the display of calorie information, reference information and staff training. These criteria were drafted by the Food Standards Agency and agreed with participating companies at the first meeting of the Calorie Labelling Group (a group formed of the companies participating in the scheme). The Food Standards Agency provided wording for reference information that companies could use in their materials, and provided information for staff to use in training in the form of a poster available to download from the FSA website. Advice was also given to companies around a number of practical issues including how to obtain calorie information, how to account for the variance of calorie values and the approach to calorie labelling taken by enforcement bodies. | Food Standards Agency and catering companies |
| Non-award 32  Unclear  Unclear | To provide healthier alternatives and change cooking techniques to provide a lower **fat**, lower **salt** product without impacting on the profitability of the business | Provision of advice pack on reformulation of recipes (reducing salt and fat), improved cooking techniques to reduce fat, marketing of water and sugar free drinks. | Local City Council Public Protection involving Environmental Health Officers and Trading Standards  City Council Principal Health Improvement Office |

*Nutrient/food group targets highlighted in* ***bold*** *(multiple nutrient/food group targets were assumed when general terms such as ‘healthy food’ or ‘nutritional quality’ were used)*

^1^The specific food outlets included were those that, as their main business, sold ready-to-eat meals and were openly accessible to the general public.

2Overall HCC criteria: Essential criteria 3: Polyunsaturated or monounsaturated **fat or oil** used for cooking. 12: Customers can add own **salt**: Sachets or salt shakers with fewer holes available. 14. Where soft drinks are sold **water**, reduced **sugar**/diet drinks and/or **unsweetened fruit juice** are available and are more prominently displayed. 21: Smaller portions are available for children and adults if requested. For businesses involved in deep fat frying. 5: Cooking oil in deep fat fryers heated to optimum temperature. 6: Excess **fat** drained from food before serving. 7: Oil in fryer is properly maintained

Bagwell, S. 2014. Healthier catering initiatives in London, UK: an effective tool for encouraging healthier consumption behaviour? *Crit Public Health,* 24**,** 35-46.

Hanratty, B., Milton, B., Ashton, M. & Whitehead, M. 2012. ‘McDonalds and KFC, it's never going to happen’: the challenges of working with food outlets to tackle the obesogenic environment. *J Public Health,* 34**,** 548-554.

Holdsworth, M., Haslam, C., Raymond, N. T. & Leibovici, D. 1997. An evaluation of the Heartbeat Award Scheme in public eating places: the customer's perspective *J Nutr Educ,* 29**,** 231-236.

Warm, D. L., Rushmere, A. E., Margetts, B. M., Kerridge, L. & Speller, V. M. 1997. The Heartbeat Award Scheme: an evaluation of catering practices *J Hum Nutr Diet,* 10**,** 171-179.
