# Supplementary material for: A description of interventions promoting healthier ready-to-eat meals (to eat in, to take away, or to be delivered) sold by specific food outlets in England: a systematic mapping and evidence synthesis
Source: BMC Public Health. 2017 Jan 19;17:93. doi: 10.1186/s12889-016-3980-2 (PMC5244522; doi:10.1186/s12889-016-3980-2)
Supplement: Additional file 7: — Description of the design, methods and results of evaluations of interventions to promote healthier ready-to-eat meals (to eat in, take away, or delivered) sold by specific food outlets in England (Tier 2, n = 30). (DOCX 63 kb) [file 12889_2016_3980_MOESM7_ESM.docx]

**Additional file 7: Description of the design, methods and results of evaluations of interventions to promote healthier ready-to-eat meals (to eat in, take away, or delivered) sold by specific^1^ food outlets in England** (Tier 2, n=30)

| **Category, project reference and type of food outlet^2^** | **Study design/**  **timing of data collection** | **Methods** | **Evaluation results** |
| --- | --- | --- | --- |
| Award 2  Takeaway eateries (1) | Cross sectional study/  Baseline measures only | Questionnaires to takeaway owners and their customers to establish interest in the intervention | **Process:** No information |
|  |  |  | **Acceptability (outlets and customers):** Feedback, on the whole, was positive with some negative consumer comments along the lines of “nanny state” and no one should interfere with chips. |
|  |  |  | **Cost:** No information |
|  |  |  | **Outcome/impact:** No information |
| Award 6  Takeaways and Sit in eateries (1, 2 and 3) | Cross sectional study/  Post intervention measures only | Survey of businesses who received the award conducted in 2010 (n=33 of 72) and 2012 (n=32 of 61) | **Process:** No information |
|  |  |  | **Acceptability (outlets):** 82% (2010) and 78% (2012) of respondents said they experienced none or very few difficulties when taking part in the scheme. 82% (2010) and 94% (2012) of businesses said they would promote the scheme to others. Benefits reported: greater recognition for businesses, which related to customer satisfaction and confidence, publicity and gaining more customers; and benefits to staff health and increasing their awareness. Difficulties reported included increased food wastage, sourcing alternative food products, and staff training issues. Staff training issues related to improving staff awareness and practices relating to promoting healthier ready-to-eat meals, including the challenges in changing the amount of salt used by chefs. |
|  |  |  | **Cost:** No information |
|  |  |  | **Outcome/impact:** No information |
| Award 10  Takeaways and Sit in eateries (1, 2 and 3) | Cross sectional study/  Post intervention measures at two time points (different data) | Questionnaires (initial questionnaire n=17; renewal questionnaire n=27) | **Process:** Only one business chose not to renew their award |
|  |  |  | **Acceptability (outlets):** Initial questionnaires (completed by new businesses): The most popular reason for applying was ‘Public Recognition’ followed closely by ‘Customer Satisfaction’. Renewal questionnaires: Only one business commented that healthy options didn’t seem to be doing that well with their customers and didn’t see them as a priority for their business so chose not to renew their award |
|  |  |  | **Cost:** No information |
|  |  |  | **Outcome/impact:** No information |
| Award 15  Takeaways and Sit in eateries (1, 2 and 3) | Cross sectional study/  Post project measures  and  Case studies | Feedback questionnaire sent to businesses (3-6 weeks after award; n not reported)  Case-studies – Anecdotal feedback (n=2) | **Process:** No information |
|  |  |  | **Acceptability (outlets):** Feedback questionnaires: inconvenience on the businesses had been minimal. |
|  |  |  | **Cost:** *Case study 1* – the owner reported increased profit margins, with less waste and better products. *Case study 2* – a fish and chip shop owner was keen to take part in the project but was a bit reluctant to use the five hole salt shakers. After only a week they asked for another five hole shaker and reported a 66% reduction in the amount of salt he was using. |
|  |  |  | **Impact/Outcome:** *Case study 1* – a takeaway, which was offering fish and chips, doner kebabs and a variety of curries, samosas etc., removed the doner and curries and concentrated on fish chips and more traditional fare. They offered grilled fish and introduced grilled homemade burgers and fish cakes, and they also introduced a choice of portion sizes. |
| Award 20  Takeaways and Sit in eateries (1, 2 and 3) | Cross sectional study/Post project measures | Feedback from businesses:   - Visit and face to face interviews with award winners (n=20) - Telephone interviews with businesses who did not apply for the award (n=36) | **Process:** no information |
|  |  |  | **Acceptability (outlets):** *Feedback from businesses with award:* Only one business felt that the scheme had not been worthwhile, with the rest responding it had either been “very worthwhile” or “quite worthwhile”. Most businesses found it either very easy or reasonably easy to meet the award criteria. Most businesses said that changing to a healthier type of oil was the easiest criteria to meet. For example those previously using a partially hydrogenated vegetable oil or saturated fat had changed to rapeseed oil after discussion with the project team. Recognition and a desire to serve healthier food were the most popular reasons for applying for the award. *Feedback from businesses without award:* The main reasons for not applying were: they would only be interested if they qualified for a gold award; not enough time; lost the information; a colleague may have been given the information and didn’t pass it on. |
|  |  |  | **Cost:** Six businesses thought there had been an increase in food sales since gaining an award, but 13 didn’t think there had been a change and one wasn’t sure. |
|  |  |  | **Outcome/impact:** No information |
| Award 25  Takeaway eateries (1) | Cross-sectional study post intervention with follow-up/6 months and 3 years) | Visits or contact with businesses (6 months follow-up n=10; 3 year follow-up n=20) | **Process:** After three years, four businesses had closed down and three had changed hands. 13 businesses were still trading as the same business; however, a number had experienced changes of staff or new managers since the project began. As a result, staff mainly had very little memory of the intervention. |
|  |  |  | **Acceptability (outlets):** The main criticism at follow-up was that the initiative had come to an end (no longer any resourcing) and businesses wanted some ‘professional’ looking posters and award certificate, rather than the budget ones that had been sent out. |
|  |  |  | **Cost:** No business experienced any noticeable loss in profits. |
|  |  |  | **Impact/outcome:** After six months all businesses were still following the award minimum criteria they had agreed to. After three years, the 16 businesses still operating were still complying with the agreed criteria. There was evidence of more creativity around use of lower fat cheeses, yogurt and salads in sandwiches; also more types of bread being used and a larger range of healthier options in relation to snacks and drinks (e.g. fruit pots, smoothies). |
| Award 26 ([Holdsworth et al., 1997](#_ENREF_3), [Warm et al., 1997](#_ENREF_4))  Takeaways and Sit in eateries (1, 2 and 3) | 1. Cross sectional study with control/Post project measures (with control/non-award group)  2. Cross sectional study/Post project measures | 1. A postal questionnaire (for both premises with and without the award): Questionnaires sent to 497 award premises and 495 controls (identified by local EHOs and broadly match to award premises).   Response rate: award = 77% (n=380); controls = 62% (n=306) (39% award and 44% control respondents were public eating places)   1. A self-completion, structured questionnaire for customers of public eating places receiving the award (n=271 of 377 approached) | **Process:** No information |
|  |  |  | **Acceptability (customers):** 53% of the customers (n = 143) were unaware that the establishment had received the award. Of the 127 (47.6%) who knew about the award, the vast majority (n = 117; 92.3%) said that they did not choose the establishment because it had an award. The majority of customers (n = 209; 78.0%) believed that the establishment offered healthy meals and snacks. The main influences on choice of eating place were quality of food (n = 195; 72.2%), affordable prices (n = 162; 60%), good service (n = 144; 53.3%), variety of choice (n = 131; 48.5%), and location (n = 114; 42.0%). The availability of healthy food choices (n = 82; 30.0%) was least often selected. However, the majority of respondents (n = 205; 78.9%) either agreed or strongly agreed that healthy food choices should be available when eating out. Just over two-thirds customers (n = 183; 67.5%) were unaware of all three main criteria of the award, while 88 (32.5%) understood correctly. Most customers (n = 224; 82.7%) were unaware that menus had been assessed by a dietitian. Thirty-nine respondents (14.4%) incorrectly believed that the award implied that all food choices were healthy choices. More than 90% of respondents correctly answered questions concerning current nutrition guidelines for reducing fat, fried food, sugar, sweets, and chocolates and increasing fruit and vegetables, although there was still confusion over whether starchy food consumption should be increased, with just over half of respondents (n = 136; 52.5%) answering correctly. |
|  |  |  | **Cost:** No information |
|  |  |  | **Impact/outcome:** *Perceptions of changes in award premises since gaining the award (% that answered ‘yes’)*: 41% more customers now used premises; 70% customers were now offered more ‘healthy’ meals (most premises already met criteria and did not have to change purchasing or cooking practices). *Foods purchased*: Award premises purchased significantly more brown rice (p<0.001), semi/skimmed milk (p<0.05) and low-fat/high polyunsaturated spreads (p<0.05). Controls purchased significantly more whole milk than Award premises (p<0.05). *Purchasing options offered*: Significantly more Award premises offered a ‘healthy’ option on menu (p<0.001); skinned chicken before cooking (p<0.05), offered potato alternative to chips/roast potatoes (p<0.05) and had fruit available as a dessert (p<0.001), compared with controls |
| Award 27 ([Hanratty et al., 2012](#_ENREF_2))  Takeaways and Sit in eateries (1, 2 and 3) | Cross sectional study/Post project measures | In-depth qualitative interviews with LA and PCT workers (n=36)  Data were analysed using the constant comparative method | **Process:** No information |
|  |  |  | **Acceptability (intervention project team):** *Experience of working on project*: PCT workers felt that they had little leverage with restaurants that were part of a chain or large commercial group; found certain skills were important to build good relationships with businesses (e.g. found it very useful that they appointed a former chef to implement the award who knew the industry and what was possible); the project was often a low priority to the individual responsible (other work commitments had higher priority) and the post was vacant for a time; it was very labour intensive working with commercial sector organisations; and consumer brand awareness in relation to the Award was poor.  Main finding: *“The public health staff knew how difficult it could be for businesses in disadvantaged areas to generate profits and were sensitive to the potential conflict between health and commercial success. Working with cafes, restaurants and takeaway food outlets was perceived to be time consuming, with many alternative areas of public health work offering better opportunities for health gain. The two awards that they were implementing, Eat Well and Breastfeeding Welcome, faced a major challenge from local culture in an area where bottle-feeding for infants and fast food were the norm. At the time of the study, the planning powers to restrict the opening of fast food outlets had not been used”.* |
|  |  |  | **Cost:** No information |
|  |  |  | **Impact/outcome:** No information |
| Award 29  Takeaways and Sit in eateries (1, 2 and 3) | Before/after study/Pre and post project measures | Case-study based evaluation (77% of the 155 businesses) including nutritional analysis of food samples  Customer interviews (n=200) | **Process:** No information |
|  |  |  | **Acceptability:** No information |
|  |  |  | **Cost:** No information |
|  |  |  | **Impact/outcome (outlets):** Energy reduction across awarded venues = 207 kcal per meal; Fat reduction 6.8g per meal; Saturated fat reduction 4.0g per meal; Sugar reduction 2.4g per meal = Added sugar reduction likely to be higher but difficult to measure; Salt reduction 0.7g per meal.  Takeaway food sector: In pre and post intervention sampling of standard portions of battered fish and chips - Typical reduction in fat content and saturated fat content by 27%.  **Impact/outcome (customers):** 28 were aware of the award. 114 (57%) selected healthier choices, 62 (54%) of which said they were influenced by the fact it was a ‘Healthier Choice’. 86 (43%) selected regular choices, of which 53 (62%) said they would consider a ‘Healthier Choice’. A ‘Healthier Choice’ was chosen by 31.5% of customers over the age of 40 and 26% of customers under the age of 40. 52 male and 62 female customers selected ‘Healthier Choices’. |
| Award 30  Takeaways and Sit in eateries (1, 2 and 3) | Before/after study/Pre and post project measures | Pre- and post intervention questionnaires for customers (number unspecified) | **Process:** No information |
|  |  |  | **Acceptability:** No information |
|  |  |  | **Cost:** No information |
|  |  |  | **Impact/outcome (customers):** *Awareness of healthy options:* over 50% of café customers in all the cafés reported awareness of healthier options. *Healthy eating levels (% eating 5 a day):* Café 1 - no change (38% pre and post-intervention); Café 2 - increase from 18% to 28%; Café 3 – increase from 20 to 27%; Café 4 - increase from 11% to 17%. Uptake of healthier options: Cafés 1 and 2 – 6% increase; Café 3 – 27% increase; Café 4 – 25% increase |
| Award 34 (HCC) ([Bagwell, 2014](#_ENREF_1))  Takeaways and Sit in eateries (1, 2 and 3) | 1. Cross sectional study/Post project measures  2. Before/ after study/  Some before and after measures (qualitative) | 1. Online survey designed to capture business data  Interviews with those responsible for implementing the scheme at a local level  2. In-depth qualitative study in three London boroughs: detailed interviews with businesses and customers, and focus groups with the EHOs, nutritionist, and public health practitioner involved in implementing the scheme (before and after implementation) | **Process:** No information |
|  |  |  | **Acceptability (outlets):** The most problematic criterion was stopping adding salt to water when cooking. Businesses reported fear that customers would not like the taste of food cooked without any salt; that wholegrain varieties of carbohydrates and fresh fruit are not popular; and lower fat spreads and mayonnaise are difficult to access from the suppliers they use. Businesses were particularly resistant to change where they fear customers might not like the taste of healthier options which will reduce sales and threaten profits. Changes are more likely to be made when they are imperceptible and not noticed by customers. |
|  |  |  | **Cost:** No information |
|  |  |  | **Impact/outcome (outlets):** Most businesses were already meeting a large number of the criteria before engaging with the HCC scheme. 63 of the 77 businesses (82%) were already meeting one of the key essential criteria: cooking with a healthier variety of oil prior to intervention. 21 businesses (27%) did not need to make any changes at all to secure the HCC award and 46 (60%) businesses adapted their practices to meet the award criteria. The number of criteria met by the businesses when awarded the HCC ranged from 9 to 23 and averaged 15. The number of changes businesses had to make to get the award ranged from 0 to 10 with an average of 2.56 changes.  The intervention appeared to have had most impact in encouraging businesses to promote healthy eating and allowing customers to add their own salt. 15 businesses started offering smaller portions for children and the 13 started selling reduced sugar drinks. 15 businesses agreed to ensure that their cooking oil was maintained at the correct temperature (essential criteria for businesses involved in frying food). |
| Award 40 (HCC)  Takeaways and Sit in eateries (1, 2 and 3) | 1. Before/ after study/ Pre and post project measures (qualitative)  2. Cross sectional study/Post project measures | 1. Focus groups with the EHOs and nutrition team (at start of and after implementation)  A review of HCC application and criteria forms to ascertain the changes made by each business (n=11)  2. Face to face interviews with businesses (n=11)  Customer survey (n=28) | **Process:** No information |
|  |  |  | **Acceptability (project team):** The training provided to those administering the scheme was found to be useful and EHOs felt confident in their ability to promote the scheme and assess businesses against the criteria. The strategy of working with a small independent chain involved a significant initial investment of time, but the changes encouraged impacted on 11 of the business’s outlets suggesting that this approach is likely to be a more resource efficient approach in the longer term.  **Acceptability (outlets):** EHOs’ supportive approach was welcomed by businesses and was key to business engagement. The businesses generally found the scheme easy to understand and appreciated the fact that the changes required of them were fairly minor and incurred little or no extra cost.  **Acceptability (customers):** The responses from customers suggested that the introduction of the HCC and the healthier changes made to menus and cooking practices as a result were not likely to radically change customers eating habits. 23 of the 28 customers surveyed stated that the changes made by the business were not likely to encourage them to eat a healthier diet at home or when eating out elsewhere. |
|  |  |  | **Cost:** The changes made by businesses incurred little or no extra cost |
|  |  |  | **Impact/outcome:** The 11 businesses surveyed for the evaluation made between zero and eight changes to achieve the award with an average of four changes each. Changes made to the use of oil and salt, which were adopted by the largest number (n=5) of businesses are particularly important. |
| Award 41 (HCC)  Takeaways and Sit in eateries (1, 2 and 3) | Cross sectional study/Post project measures | Unclear | **Process:** No information |
|  |  |  | **Acceptability (outlets)** Several of the business indicated that the HCC helped them jump start changes in their establishments and helped make them aware of simple ways to make significant changes:  *“The pledge showed that even in traditionally the unhealthiest of eating environments, improvements can be made in eating habits. Spectators can mirror the healthy eating habits of the sports people they watch.”* – Football Club, *“Made me and the catering staff that I work with think about what we were doing and how we could improve this for our customers health.”* – YMCA |
|  |  |  | **Cost:** No information |
|  |  |  | **Impact/outcome:** 43% of 42 business said they are selling more water and diet drinks now they are prominently displayed; 14% of the businesses reported their customers have been asking for smaller portions now they are clearly advertised; 85% of businesses agreed that the HCC lead them to make further healthier changes in their establishment. |
| Award 42 (HCC)  Takeaways and Sit in eateries (1, 2 and 3) | Cross sectional study/Post project measures | Changes made by businesses – unclear (n=9)  Business feedback – questionnaire/interview? (n=9)  Customer survey (n=40) | **Process:** No information |
|  |  |  | **Acceptability (outlets):** None of the owners were aware of HCC before they were engaged as part of this project; all of the owners said using the HCC menu and posters made it easy to find the healthier options available on the menu; all owners said HCC clearly describes its aim, to provide some food and drinks that are healthier and more balanced; All owners thought that their customers were either interested or partially interested to try a more balanced option now it is available on the menu; owners described HCC as a guide, interesting and motivating as their most popular words  **Acceptability (customers):** Customers awareness of HCC (10 out of 40), customers’ ability to use materials as guide to find healthier options (35 identified five or more), ease of use (33 found easy) and intention to use to guide choices (all). |
|  |  |  | **Cost:** No information |
|  |  |  | **Impact/outcome:** Four businesses were already meeting criteria and made no further changes. Other businesses implemented a variety of changes e.g. using five hole salt shaker, advertising smaller portions, using thermostat to check oil temperature, swopping palm oil to rapeseed oil. 80% reported that more than five balanced (healthier) options were available to purchase at their business, with some having over 10 options. |
| Award 43 (HCC)  Takeaways and Sit in eateries (1, 2 and 3) | Cross sectional study/Post project measures | Unclear – observations of businesses achieving award? Anecdotal feedback from businesses? | **Process** 23 businesses achieved award out of 60 businesses invited. Almost half of restaurants and cafes achieved award (17 of 37) compared with only a quarter of the takeaways (six of 23). |
|  |  |  | **Acceptability (outlets):** Reasons for higher achievement in restaurants and cafes, compared with takeaways include: Cafes usually offer a provision broadly acceptable to HCC criteria; restaurants have a broader often ‘healthier’ provision available for customers to chooses from; a number of cafes and sandwich bars do not fry foods as standard; takeaways often do not fry in an acceptable oil type and broadly will not change their oil type; takeaway owners tend to be less willing to change their practices or are very dismissive of any engagement with HCC. Businesses near schools unwilling to join HCC. “Unless I’m forced to by the Government, I’m not going to change a thing” – manager of ‘popular school child frequented’ chicken shop |
|  |  |  | **Cost:** No information |
|  |  |  | **Impact/outcome:** No information |
| Non-award 9  Takeaways and Sit in eateries (1, 2 and 3) | Cross sectional study/Pre-intervention measures | Unclear – interviews or anecdotal evidence from businesses? (n not reported) | **Process:** The importance of building rapport and relationships of trust between the takeaway community and the support community cannot be emphasised enough. This enabled the sharing of recipes (often family recipes which are ‘secret’), and sharing of ideas and kitchen practices. If this rapport had not existed with outlet owners and project team, the project would never have been possible.  Takeaway outlets, in general, do not document recipes. This can result in inconsistency in meals, and possibly chefs using their own recipes and techniques to prepare meals – some outlets have more than one chef, resulting in possibly several methods/recipes used to prepare the same meal.  Some dietary analysis software may be inappropriate in the analysis of ingredients in the takeaway food industry – many common ingredients used in Chinese and Indian ethnic recipes are not listed in the software. |
|  |  |  | **Acceptability:** No information |
|  |  |  | **Cost:** No information |
|  |  |  | **Impact/outcome:** No information |
| Non-award 15  Takeaways and Sit in eateries (1, 2 and 3) | Before/after study/Measures at baseline and six months | Nutritional analysis of food samples (n=11)  Record of progress (assessor notes; n=11)  Sales and stock purchasing data (n=11)  Customer questionnaire (n not reported) | **Process:** No information |
|  |  |  | **Acceptability (outlets):** Top Tips successfully implemented: Using lower fat mayonnaise; Using lower fat spread if requested, otherwise none; Offering brown or granary bread; Using less oil; Removing visible fat from bacon; Using portion control; Draining food on kitchen roll before service; Removing fat from the griddle regularly; Removing excess fat from the bottom of the bain marie. Top Tips difficult to implement: Offering fresh fruit and fruit juice; Offering a healthy meal deal; Use of mature cheddar cheese (stronger flavour so less is used). Nine businesses said project did not involve any extra time.  **Acceptability (customers):** In four businesses customers liked the healthy options: soups, jacket potatoes, brown bread and fruit. In two businesses customers did not like less salt in mashed potato. One customer did not agree with fruit being sold. |
|  |  |  | **Cost:** No business saw a loss in profit. Many saw increase in sales of cold sandwich fillings. Case study premises found customer base increased due to sale of healthier meals. |
|  |  |  | **Impact/outcome:** There were decreases in fat, salt and calorie content between samples (e.g. bacon and egg sandwich 480kcal reduced to 389kcal, 16.8g fat reduced to 10.7g fat, and 4.1g salt reduced to 3.9g). |
| Non-award 16  Takeaways and Sit in eateries (1, 2 and 3) | 1. Cross sectional study/Post project measures 2. Before/ after study/ Pre and post project measures | 1. Questionnaire (completed by officer during 2^nd^ visit)  Data collection card (completed by business after 4 weeks – products purchased and their cost and the total sales for that week)  Customer Questionnaire  Business Questionnaire (completed post project – feedback and comments about the project)  Officer Questionnaire (Officer feedback)  n’s not reported  2. Nutritional sampling (n=2) | **Process:** No information |
|  |  |  | **Acceptability (project team):** The aims of the project have been met. It is essential for the officer to be positive and enthusiastic about the project and to have an interest in leading a healthier lifestyle, as the proprietors will naturally be concerned about the time and financial implications of taking part in the project initially and their motivation will rise and fall throughout the project, therefore the officer will need to give lots of encouragement. Some businesses needed a lot of extra encouragement in the form of visits and telephone calls, in addition to the planned visits and, therefore, the time taken was more than initially thought and should be considered when planning workloads etc as this type of work is not always seen as a priority in all authorities. Two authorities felt that they needed to do sampling to help educate the businesses, in one case the results showed a reduction in fat but an increase in calories, these results were able to show the business the importance of portion control as the second sample was heavier.  **Acceptability (outlets):** Top tips easiest to implement: Using the lower fat mayonnaise; Using the Lower fat spread and only if requested, otherwise not using any spread; Using low salt and low sugar baked beans; Offering brown or granary bread; Using less oil; Removing visible fat from the bacon; Using portion control; Draining food on kitchen roll before service; Removing fat from the griddle regularly and; Removing excess fat from the bottom of the bain marie. These top tips were more popular with the proprietors, as their customers in all except one premise did not notice any difference in taste. The top tips that were successful and popular, varied between each business suggesting that each business is individual due to factors such as the drive of the proprietor, the customer base and the location. The top tips difficult to implement were: Offering fresh fruit and offering a healthy meal deal. These top tips were less popular as customers did not want to buy pieces of fresh fruit and fruit juice and although some proprietors have started to offer healthier lunches such as soups and jacket potatoes. They found it difficult to create a healthy meal deal option because they did not know what to include and how to price it. In all but one case the proprietors did not find it time consuming to drain the food on some kitchen roll before service or to clean the griddle regularly. The most popular piece of equipment was the small bottle or spray bottle provided for dispensing less oil on to the griddle. The proprietors liked this as it helped them to use less oil, thus saving them money as well as helping to improve their customers’ health. The proprietors also particularly liked the lower fat mayonnaise and spread.  **Acceptability (customers):** Customers did not notice any difference in taste in all except one premise. Customers liked having the option of brown and granary bread as well as white bread. Most customers did not object when asked if they would like the visible fat to be trimmed off their bacon, and in two cases positive comments were made. In all but one case the proprietors did not receive any negative comments from customers when using portion control. |
|  |  |  | **Cost:** The small bottle or spray bottle provided helped proprietor to use less oil, thus saving them money. In one case the proprietor found using a reduced fat spread was cheaper than the higher fat version and they believe that using this will save them on average £1500 per year. One businesses saw a loss in profit as a result of taking part, four businesses were unaffected by the changes, three showed a profit and two have not yet submitted this information. |
|  |  |  | **Impact/outcome:** *Case 1* – reduction in fat but an increase in calories (second sample was heavier). *Case 2* – no significant changes (due to the proprietor only changing the bread type to wholemeal otherwise the meal was the same in respect of using butter and leaving the fat on the bacon). |
| Non-award 17  Takeaways and Sit in eateries (1, 2 and 3) | 1. Before/ after study (including case-study)/Pre and post project measures  2. Cross sectional study/Post project measures | Visits to businesses (1. assessments conducted baseline and follow-up, and 2. feedback from businesses at follow-up visit) | **Process:** No information |
|  |  |  | **Acceptability (outlets):** The majority of the businesses were very supportive of the initiative and happy to visibly take part in the scheme, for example through displaying posters. *Positive feedback from businesses:* Using the labelling information to make healthier choices e.g. type of oil purchased or healthier margarine; Offering granary and brown bread as well as white; Using less oil, especially when an oil spray was provided and used with the griddle; Using less salt; Remove visible fat from bacon; Smaller portions provided but not necessarily advertised; Some success in using a smaller amount of cheese. Feedback from businesses was generally very positive and they found the intervention very useful, including the information pack. One business however did not welcome the intervention and did not wish to take part after the initial visit and felt it was not an area of work that the service should be engaged in. *Other feedback:* Businesses not consistently providing fruit and salad items, especially in the colder months; Some businesses have found that unsweetened fruit juices and healthier yoghurts have not sold; Healthy meal deals have not generally been implemented; Businesses not always good at promoting healthier options; Higher meat content sausages were too expensive or customers did not like the taste; Specific healthier alternatives (e.g. tuna in spring water) are not always available in wholesale establishments. |
|  |  |  | **Cost:** Some of the business saw an increase in sales, including healthier products. Other businesses have shown no change in sales. |
|  |  |  | **Impact/outcome:** A number of small changes were made by most caterers thus becoming part of the proprietor’s daily routine. Some businesses also started to provide smaller portions, or advertise them better. *Case-study results:* 50% less salt used since the first visit; 50% less oil used (oil now purchased every two weeks instead of every week) and the business operator is using a spray; 50% less cheese now used since changing to a stronger type now 1kg is purchased every two weeks; Fruit and yoghurt are now provided and sales are good; Granary bread is now sold, one in five customers taking this option; Smaller portions are now available and advertised. |
| Non-award 18  Takeaways and Sit in eateries (1, 2 and 3) | Cross sectional study Post project measures | Business Questionnaire (n=2) | **Process:** No information |
|  |  |  | **Acceptability (outlets):** Both businesses were very keen and positive in their response to the project but individual results for these businesses not reported. |
|  |  |  | **Cost:** No information |
|  |  |  | **Impact/outcomes:** No information |
| Non-award 21  Takeaways and Sit in eateries (1, 2 and 3) | 1. Before/ after study/ Pre and post project measures  2. Cross sectional study/post project measures | 1. Visits to business (n=22)  2. Feedback questionnaire (training component; n not reported) | **Process:** No information |
|  |  |  | **Acceptability (outlets):** Feedback on the training was 94% positive |
|  |  |  | **Cost:** No information |
|  |  |  | **Impact/outcomes:** Only four of the 22 businesses visited made no changes to their menus. Changes in the remaining 18 businesses included changing menus completely, removing their children’s menu and offered smaller portions of the adult menu instead, and making small changes to a number if their recipes across the whole menu as well as changing portion sizes. |
| Non-award 23  Takeaway eateries (1) | Cross sectional study/Post project measures | Nutritional analysis but methods unclear – food samples analysed or recipes inputted into nutritional analysis software?  Sales records  Feedback but methods unclear – structured surveys/interviews or anecdotal feedback? | **Process:** No information |
|  |  |  | **Acceptability (customers):** Customers were enthusiastic about the taste of the food, describing it as “delicious”, “so good” and “the best thing”. Students commented that they liked the availability of an alternative to other fast food outlets, saying “There are only dodgy places around otherwise” and “It’s really good, convenient and we like it a lot.” Many instances where people made the decision to buy Box Chicken in preference to fried chicken from fast food outlets were recorded. |
|  |  |  | **Cost:** No information |
|  |  |  | **Impact/outcome:** The menu met the Department of Health’s recommendations around sugar, salt, saturated fat, calories and fruit/vegetable portions. The trader was able to serve a meal that contained a tenth of the saturated fat, less than a fifth of the salt, and fewer than half the calories, plus included an extra 2.5 portions of fruit and vegetables, compared to a meal at KFC. |
| Non-award 24  Takeaway eateries (1) | 1. Before/ after study/ Pre and post project measures  2. Cross sectional study/Post project measures | 1. Visits to businesses  2. Verbal evaluation (impact of changes and any customer feedback) | **Process:** No information |
|  |  |  | **Acceptability (outlets and customers):** Salt shaker not popular in some outlets. Complaints re lack of taste and longer queues due to it taking longer to salt! |
|  |  |  | **Cost:** No information |
|  |  |  | **Impact/outcome:** Outlets took on board many of the recommendations made, with the exception of those they perceived would not have sold well. All outlets added healthier drinks to their drinks selection, and some of the restaurants added their own further ideas for healthier options. |
| Non-award 25  Takeaway eateries (1) | 1. Before/ after study/ Pre and post project measures (sub-sample)  2. Cross sectional study/Post project measures | 1. Nutritional analysis of food samples (baseline and 12 month follow-up; n not reported)  2. Visit to business – catering practices (at 12 months; n=50) | **Process:** No information |
|  |  |  | **Acceptability:** No information |
|  |  |  | **Cost:** No information |
|  |  |  | **Impact/outcome:** 25% reduction in salt levels. Little change in fat. Use of salt shakers - good uptake – only four no longer used these; only 6% businesses added salt to batter; no businesses added salt to gravy and curry; of the 30% who added salt to peas at start, the majority had stopped or reduced amount added; two businesses sources lower salt gravy during project; only three businesses used rapeseed oil or oils lower in saturated fat; a few businesses had adopted one or more of the food preparation methods to reduce fat; 10% of premises started to use low fat mayonnaise during project and 32% changed to low fat spread; Drinks – unclear whether this had changed as it looks as if most premises sold water at same or lower price than carbonated drinks |
| Non-award 26  Takeaway eateries (1) | 1. Before/ after study/ Pre and post project measures  2. Cross sectional study/Post project measures | 1. Baseline survey completed during visit to business (n=23)  1 & 2. Follow-up postal survey completed by businesses (n=30) | **Process:** No information |
|  |  |  | **Acceptability (outlets):** Businesses responded positively to a role as custodians of customer health.  **Acceptability (customers):** The project also generated consumer debate and raised awareness of salt reduction and health. Some fish and chip shops reported some negative reaction by customers. This has prompted a debate about whether a lower profile, less publicised approach, often described as “health by stealth” may have been more effective. |
|  |  |  | **Cost:** There has been a significant reduction in salt usage at local fish and chip shops, providing a cost saving to the businesses. |
|  |  |  | **Impact/outcome:** 97% of businesses using the 5 hole salt shakers (just one business not using; 100% used 17 hole shaker at baseline). All of the businesses using the five hole shakers also displayed the poster advertising the campaign. Most customers continued to apply salt to their food (appears to be no different to baseline). Number of times salt shakers refilled (baseline to follow-up): 9% to 0% >1x per day, 28% to 24% 1x per day, 27% to 24% 3-4x per week, 18% to 33% 2x per week. |
| Non-award 27  Takeaway eateries (1) | Cross sectional study/Post project measures | Success of the scheme was primarily to be judged by acceptance of the new shakers by the shops  Anecdotal feedback | **Process:** Only three businesses that were approached declined to take part. A large proportion of the shops agreed to provide a poster and leaflets saying why the new shakers were being introduced. |
|  |  |  | **Acceptability (outlets):** The feedback from the businesses was extremely positive. |
|  |  |  | **Cost:** One of the businesses took a record of the impact on salt consumption (from orders with the wholesaler) and advised that this was down by 60%). |
|  |  |  | **Impact/outcome:** One of the businesses took a record of the impact on salt consumption (from orders with the wholesaler) and advised that this was down by 60%). |
| Non-award 28  Takeaway eateries (1) | Before/after study with control/pre and post project measures | Pre and post-questionnaires, completed during visits to businesses by project nutritionist  (n=2 businesses participated, n=1 business acting as a wait list control) | **Process:** No information |
|  |  |  | **Acceptability (outlets):** Both shops were very enthusiastic about the project. One business reported finding the changes easy to manage. |
|  |  |  | **Cost:** Both shops reported a financial gain during the intervention period and predicted a future saving. One business reported reduced food waste. |
|  |  |  | **Impact/outcome:** Both shops successfully implemented healthier changes to their sandwiches. These included things such as: Switching to a spread lower in saturated fat; Switching to a mayonnaise lower in saturated fat; Reducing the portion sizes of fillings; and Offering healthy meal deals at a reduced price. One business reported a better knowledge of nutrition. |
| Non-award 29  Takeaway eateries (1) | Cross sectional study/Post project assessment | The county Food Partnership reviewed the work on the project post intervention but methods unclear | **Process:** No information |
|  |  |  | **Acceptability (project team):** The Partnership concluded that, taking into consideration the resources spent and the impact the project had had, and the project merited taking further. The officers agreed to carry on with the project in order to try and recruit more sandwich manufacturers onto the scheme.  Lessons learnt:  1. Start small – work with the smaller sandwich manufacturers. The majority of all supermarkets already offer healthy options anyhow.  2. Ask businesses what they want – posters, training seminars, etc  3. As with all things – it takes longer than you think!  4. Well worth getting the right people together – has proved very beneficial in other areas of work also.  5. Each profession found that this work linked well with their services’ objectives. |
|  |  |  | **Cost:** No information |
|  |  |  | **Impact/outcome:** So far the Partnership are aware of eight out of 84 sandwich manufacturers offering healthier options however due to the campaigning many more may have taken the advice on board without the Partnerships knowledge. |
| Non-award 30  Takeaways and Sit in eateries (1, 2 and 3) | Cross sectional study/Post project measures | Interviews with customers (n=50) | **Process:** No information |
|  |  |  | **Acceptability (customers):** 20% of customers said they were already aware of the project and 96% were able to identify balanced options when shown a takeaway menu and the project poster. 92% said that the objectives of the project were clearly described and indicated that the manner in which it was presented was clear enough to be understood. 52% found it easy to find the healthier options available on the menu. 84% were interested in trying a more balanced option when they next ordered. Those interviewed chose the words ‘different’, ‘helpful’ and ‘useful’ when asked to describe the project. The positive choice of words reflected a favourable acceptance of the project by customers. The results indicate that one of the key barriers to behaviour change had been overcome – customers’ ambivalence to change. |
|  |  |  | **Cost:** No information |
|  |  |  | **Impact/outcome:** No information |
| Non-award 31  Takeaways and Sit in eateries (1, 2 and 3) | Cross sectional study/Post project measures | Interviews with company representatives, outlet managers to evaluate the process of implementation (n=39)  Point of choice and post choice interviews carried out with customers (n=289)  Focus groups with customers of the outlets in the scheme (n=8 groups, total n not reported) | **Process:** No information |
|  |  |  | **Acceptability (outlets):** Businesses were able to implement the calorie information scheme and any issues encountered along the way were overcome with relative ease. *Key learning points from the businesses’ perspective:* the process of setting up and rolling out the scheme worked better where there was close liaison between head offices and outlets; business systems, such as IT programs, could cause problems; plenty of time for planning and setting up was needed, as some things took more time than expected; there was a need for businesses to consider the design of materials, so that they could be used in all of their outlets.  **Acceptability (customers):** Three factors were found to have an impact on customers’ capacity and inclination to use calorie information: Visibility – Calorie information is most visible when its size, colour and location allow it to stand out from other information, including price and product descriptions; Understanding (there were three particular knowledge gaps which impeded understanding: Calories so closely associated with dieting for weight loss that people could not see a wider application for them; lack of understanding on recommended daily allowances for calorie intake; lack of knowledge about how much exercise was needed to burn off calories, particularly amongst people in manual jobs, who felt they needed highly calorific meals to enable them to undertake their work); Consumer engagement – preconceptions about diet and healthy eating affected people’s inclination to use calorie information. Positive messages and tips on saving calories were effective in engaging consumers. Consumers could envisage using calorie information to enable them to make positive food choices and to maintain a healthy weight whilst still enjoying their favourite foods and their favourite food outlets. Although the actual usage of calorie information in catering outlets was low, it is important to note that the scheme had only been in place for a relatively short time and people were not expecting to see calorie information. Consumers suggested that the impact of calorie information on food choice would increase over time as awareness of calorie information was likely to increase. Consumers may, therefore, become more engaged with CI as they learn to expect to see it. |
|  |  |  | **Cost:** No information |
|  |  |  | **Impact/outcome:** Menu choices were influenced by the calorie information for just a small number of customers. |

^1^The specific food outlets included were those that, as their main business, sold ready-to-eat meals and were openly accessible to the general public.

^2^Food outlets targeted by the intervention were mapped (see Box 1 for detail of process) onto one of three categories:

1. Takeaway eateries (takeaways)
2. Sit-in eateries
3. Food outlets that included options to takeaway or sit-in

Bagwell, S. 2014. Healthier catering initiatives in London, UK: an effective tool for encouraging healthier consumption behaviour? *Crit Public Health,* 24**,** 35-46.

Hanratty, B., Milton, B., Ashton, M. & Whitehead, M. 2012. ‘McDonalds and KFC, it's never going to happen’: the challenges of working with food outlets to tackle the obesogenic environment. *J Public Health,* 34**,** 548-554.

Holdsworth, M., Haslam, C., Raymond, N. T. & Leibovici, D. 1997. An evaluation of the Heartbeat Award Scheme in public eating places: the customer's perspective *J Nutr Educ,* 29**,** 231-236.

Warm, D. L., Rushmere, A. E., Margetts, B. M., Kerridge, L. & Speller, V. M. 1997. The Heartbeat Award Scheme: an evaluation of catering practices *J Hum Nutr Diet,* 10**,** 171-179.
